# SNP‐ and haplotype‐based single‐step genomic predictions for body weight, wool, and reproductive traits in North American Rambouillet sheep

**DOI:** 10.1111/jbg.12748

**Published:** 2022-11-21

**Authors:** Andre C. Araujo, Paulo L. S. Carneiro, Hinayah R. Oliveira, Ronald M. Lewis, Luiz F. Brito

**Affiliations:** ^1^ Graduate Program in Animal Sciences State University of Southwestern Bahia Itapetinga Bahia Brazil; ^2^ Department of Animal Sciences Purdue University West Lafayette Indiana USA; ^3^ Department of Biology State University of Southwestern Bahia Jequié Bahia Brazil; ^4^ Department of Animal Sciences University of Nebraska‐Lincoln Lincoln Nebraska USA

**Keywords:** Best Linear Unbiased Predictions, genomic‐enhanced estimated breeding values, haplotype prediction, linkage disequilibrium, small ruminants, single‐step genomic BLUP

## Abstract

Rambouillet sheep are commonly raised in extensive grazing systems in the US, mainly for wool and meat production. Genomic evaluations in US sheep breeds, including Rambouillet, are still incipient. Therefore, we aimed to evaluate the feasibility of performing genomic prediction of breeding values for various traits in Rambouillet sheep based on single nucleotide polymorphisms (SNP) or haplotypes (fitted as pseudo‐SNP) under a single‐step GBLUP approach. A total of 28,834 records for birth weight (BWT), 23,306 for postweaning weight (PWT), 5,832 for yearling weight (YWT), 9,880 for yearling fibre diameter (YFD), 11,872 for yearling greasy fleece weight (YGFW), and 15,984 for number of lambs born (NLB) were used in this study. Seven hundred forty‐one individuals were genotyped using a moderate (50 K; *n* = 677) or high (600 K; *n* = 64) density SNP panel, in which 32 K SNP in common between the two SNP panels (after genotypic quality control) were used for further analyses. Single‐step genomic predictions using SNP (H‐BLUP) or haplotypes (HAP‐BLUP) from blocks with different linkage disequilibrium (LD) thresholds (0.15, 0.35, 0.50, 0.65, and 0.80) were evaluated. We also considered different blending parameters when constructing the genomic relationship matrix used to predict the genomic‐enhanced estimated breeding values (GEBV), with alpha equal to 0.95 or 0.50. The GEBV were compared to the estimated breeding values (EBV) obtained from traditional pedigree‐based evaluations (A‐BLUP). The mean theoretical accuracy ranged from 0.499 (A‐BLUP for PWT) to 0.795 (HAP‐BLUP using haplotypes from blocks with LD threshold of 0.35 and alpha equal to 0.95 for YFD). The prediction accuracies ranged from 0.143 (A‐BLUP for PWT) to 0.330 (A‐BLUP for YGFW) while the prediction bias ranged from −0.104 (H‐BLUP for PWT) to 0.087 (HAP‐BLUP using haplotypes from blocks with LD threshold of 0.15 and alpha equal to 0.95 for YGFW). The GEBV dispersion ranged from 0.428 (A‐BLUP for PWT) to 1.035 (A‐BLUP for YGFW). Similar results were observed for H‐BLUP or HAP‐BLUP, independently of the LD threshold to create the haplotypes, alpha value, or trait analysed. Using genomic information (fitting individual SNP or haplotypes) provided similar or higher prediction and theoretical accuracies and reduced the dispersion of the GEBV for body weight, wool, and reproductive traits in Rambouillet sheep. However, there were no clear improvements in the prediction bias when compared to pedigree‐based predictions. The next step will be to enlarge the training populations for this breed to increase the benefits of genomic predictions.

## 1 INTRODUCTION

The global demand for products from small ruminants is increasing. Further scientific innovation, with its greater application through increased education and training, is needed to meet this demand (Mazinani & Rude, 2020). Rambouillet is a common sheep breed in the US originating from Spanish Merinos that were first moved to a farm in Rambouillet, France, late in the eighteenth century (American Rambouillet Sheep Breeders Association). Known for producing high‐quality meat and wool (Thorne et al., 2022), Rambouillet sheep are commonly raised in extensive grazing systems under a wide range of climatic conditions. Rambouillet sheep produces heavy fleeces with fine fibre diameter (Thorne et al., 2021), which are suitable for the wool market. Yet, as a dual‐purpose breed, body weight and reproductive traits are also of economic importance in this breed (Thorne et al., 2021). Estimated breeding values (EBV) for these sets of traits have been generated and shared with US sheep producers through the National Sheep Improvement Program (NSIP; Notter, 1998). However, genomic evaluations are not yet available for the Rambouillet breed in the US.

With the availability of large‐scale genomic information, the pedigree relationship matrix (A), originally used to obtain EBV from Best Linear Unbiased Predictions (BLUP), can be replaced or combined with the genomic relationship matrix (G) to predict genomic‐enhanced breeding values (GEBV; Aguilar et al., 2010). The GEBV can be more accurate than EBV especially for young animals (not yet recorded for the traits of interest) and for lowly heritable and sex‐limited traits (Meuwissen et al., 2001). Furthermore, GEBV can provide advantages for the evaluation of difficult‐ or expensive‐to‐measure traits (Brito et al., 2020; Thorne et al., 2021).

The single‐step genomic BLUP (ssGBLUP; Christensen & Lund, 2010; Legarra et al., 2009) is a method that simultaneously includes both genotyped and non‐genotyped individuals in the analysis to obtain GEBV for all individuals by combining the genomic and pedigree information. The ssGBLUP is more compatible with current breeding programs (where not all breeding individuals are genotyped) and provides similar or better results than other methods (Guarini et al., 2018; Legarra et al., 2014). However, an important consideration when implementing the ssGBLUP method is how to weight the genomic and pedigree information (McMillan & Swan, 2017; Meyer et al., 2018). This conundrum arises because as G is based on the relationships at the genomic marker level, it can be difficult to invert, may not be on the same scale as the A, and may not account for residual polygenic effects (Meyer et al., 2018).

To facilitate the inversion process, and to account for residual polygenic effects, two parameters, α and β (with α=0,…,1 and β=1−α), are commonly used to include a proportion of A in the G that is used in the genomic evaluation (Meyer et al., 2018). Values between 0.95 and 0.99 are common choices to weight G (McMillan & Swan, 2017). However, some authors (Gao et al., 2012; McMillan & Swan, 2017) showed that different α can affect the accuracy and bias of the single‐step genomic predictions. McMillan and Swan (2017) used α = 0.50 to place equal emphasis on the pedigree and genomic relationships in Terminal‐Sire sheep breeds, due to less dispersion and similar accuracies of the GEBV compared to the use of higher α values. Therefore, defining the appropriate value for these parameters is important as they may differ even for different traits in the same population (Gao et al., 2012).

The G matrix used in the ssGBLUP can also be computed based on different methods. Fitting single nucleotide polymorphisms (SNP) has been the standard method used in genomic analyses. However, haplotypes can also be used for both genomic prediction (Araujo et al., 2021; Feitosa et al., 2020; Teissier et al., 2020) and genome‐wide association (Araujo et al., 2022; Bovo et al., 2021; Feitosa et al., 2021) studies. Haplotypes are the alleles from a set of adjacent loci (sizable regions called haplotype blocks or haploblocks) expected to be inherited together due to lower recombination (Gabriel et al., 2002). Haplotypes are also expected to be in higher linkage disequilibrium (LD) with the quantitative trait loci (QTL) than the single SNP (Calus et al., 2008) and capture epistatic effects (Hess et al., 2017; Jiang et al., 2018), which could result in higher accuracies and lower bias in the genomic predictions (Araujo et al., 2021; Calus et al., 2008).

Previous studies using haplotypes in genomic predictions in livestock have shown a varying improvement in the accuracies of genomic prediction, ranging from no to small (Mucha et al., 2019) up to 22% (Teissier et al., 2020) increases in GEBV accuracy compared to SNPs. The potential reasons for these inconsistencies are multifaceted. The first one is the genetic background of the populations. Most studies evaluating genomic predictions based on haplotypes were performed in cattle and purebred populations (e.g., Cuyabano et al., 2014, 2015; Feitosa et al., 2020; Mucha et al., 2019; Xu et al., 2020), which have relatively low effective population size (*N*_e_). Furthermore, in these studies, the factors that might contribute to better haplotype‐based predictions (e.g., epistasis) were not fully explored. Second, the method used to create the haplotypes can influence the results (Araujo et al., 2021; Hess et al., 2017). Several methods have been investigated including genomic windows with different numbers of SNP (usually in kb) and LD based, for instance, on different threshold levels (Teissier et al., 2020). The coding of the haplotype alleles and how to create the genomic relationship matrices including haplotypes also show differences among studies. Haplotypes tend to be multiallelic and can be either fitted as multiallelic markers in a Genomic Restricted Maximum Likelihood‐BLUP (GREML‐GBLUP) approach (Da, 2015; Prakapenka et al., 2020) or as biallelic pseudo‐SNP (ps‐SNP) defined based on the haplotype alleles in a ssGBLUP approach (Teissier et al., 2020). Further exploration of the potential value of using haplotype information in genomic prediction is still needed, particularly in small ruminants.

The implementation of genomic selection is still incipient in small ruminants, mainly due to the relatively high cost of genotyping per animal (Mrode et al., 2018), smaller herd sizes, and lower adoption of reproductive technologies such as artificial insemination (in comparison to cattle). Araujo et al. (2021) hypothesized that fitting haplotypes in genomic predictions could outperform the use of SNP in populations with high *N*_e_ because it would better capture the complex interactions within haploblocks. However, these authors did not simulate epistasis and recommended new studies in real populations. Sheep is a specie in which moderate to high *N*_e_ are common in commercial populations (Brito, Mcewan, et al., 2017; Kijas et al., 2012), with predictions of GEBV based on haplotypes still scarce (Araujo et al., 2021). Therefore, we aimed to evaluate the accuracy, bias, dispersion, and theoretical accuracy (TA) of GEBV using ssGBLUP fitting SNP or haplotypes (as ps‐SNP) for body weight, wool, and reproductive traits in Rambouillet sheep and make comparisons with traditional pedigree‐based genetic evaluations. We also evaluated the effect of constructing the haplotypes with different LD thresholds and using alternative α values when forming the G matrix. Finally, recommendations for future steps for the implementation of genomic evaluations in Rambouillet sheep were provided.

## 2 MATERIAL AND METHODS

No ethical review and approval were needed for this study because all the datasets used were provided by commercial breeding operations.

### 2.1 Phenotypic and pedigree data

The phenotypic and pedigree datasets were provided by the NSIP, which included three body weight traits [birth weight (BWT), postweaning weight (PWT), and yearling weight (YWT)], two wool traits [yearling fibre diameter (YFD) and yearling greasy fleece weight (YGFW)] and one reproductive trait [number of lambs born (NLB)] as described in Table 1. The BWT trait represents the lamb weight recorded within 24 h after birth, while PWT and YWT are the body weights recorded at five to 10 (151–304 days) and 10 to 14 (305–428 days) months of age, respectively. The wool traits were measured at yearling age (10–14 months). The pedigree dataset had 36,297 individuals born from 1985 to 2021, spanning up to 15 generations from animals with phenotypic records and with an average (standard deviation) Pedigree Completeness Index (PCI) of 0.57 (0.34). The number of generations traced back and PCI were calculated using the “optiSel” r package (Wellmann, 2019).

**TABLE 1:** Description of the datasets used for the genetic and genomic predictions of birth weight (BWT), postweaning body weight (PWT), yearling body weight (YWT), yearling fibre diameter (YFD), yearling greasy fleece weight (YGFW), and number of lambs born (NLB) in Rambouillet sheep

Dataset	Variable^a^	Trait					
		BWT (kg)	PWT (kg)	YWT (kg)	YFD (μm)	YGFW (kg)	NLB (count)
Complete	Average	4.86	35.36	59.99	18.87	3.04	1.71
	*SD*	1.02	7.78	15.28	1.67	0.81	0.59
	Individuals (*n*)	28,317	22,781	5653	9586	11,542	6846
	Records (*n*)	28,317	22,781	5653	9586	11,542	15,904
	CG (*n*)	427	461	110	195	210	445
	Genotypes (*n*)	587	632	442	529	502	242
Partial	Average	4.77	35.01	58.03	18,92	2.99	1.79
	*SD*	0.98	7.12	13.54	1.57	0.72	0.59
	Individuals (*n*)	22,115	17,118	3657	6808	9055	5407
	Records (*n*)	22,115	17,118	3657	6808	9055	13,790
	CG (*n*)	404	402	101	175	198	411
	Genotypes (*n*)	469	456	341	426	402	138
	Focal (*n*)	118	176	101	103	100	104

^a^ Standard deviation (*SD*); number of phenotyped individuals (Individuals), records (Records), contemporary groups (CG), and genotypes (Genotypes) included in the whole and partial datasets (after quality control); and number of focal (also known as validation) individuals (Focal). All genotyped and focal animals had own phenotypes or progeny with phenotypes. The complete data set contained all corrected phenotypes after quality control, and the partial data set was a subset of the complete data truncated by the birth year of the focal individuals (young selection candidates used to compare the methods evaluated).

The phenotypic datasets used for the genetic and genomic prediction analyses for the body weight and wool traits were processed previously by the NSIP, which provided preadjusted phenotypes (http://nsip.org/wp‐content/uploads/2015/04/Lambplan‐TC‐Report‐Notter.pdf). Briefly, the preadjustment considered birth and rearing type (fixed levels as a multiplicative adjustment), and age of dam (measured in days) at recording (quadratic and linear regressions). No preadjustments were done for NLB. The data underwent quality control (QC) with observations deviating more than three standard deviations from the mean removed from further analyses.

Contemporary groups (CG) were created by concatenating flock, year, season, management group, sex, recording date, and 70‐day age groups to split lambing (birth) dates into 70‐day consecutive periods for PWT, YWT, YFD, and YGFW. For BWT, the CG included all the effects previously mentioned for body weight and wool traits, excluding recording date; 35‐ rather than 70‐day age group were also used. The CG for the NLB were created considering ewe's flock, birth year, season, management group, and parturition number (e.g., ewe's first, second, or third lambing). The preadjusted phenotypes for body weight and wool traits and the NLB phenotypes were then adjusted for the CG effect, so that the phenotypes analysed—henceforth referred to as corrected phenotypes—accounted for all systematic environmental effects considered in the NSIP genetic evaluations. The CG for all analysed traits were treated as fixed effects and, as a final QC step, CG with less than three animals and with no phenotypic variability within CG were removed.

### 2.2 Genotypic data

Samples from 741 animals were genotyped using the GeneSeek Genomic Profiler Ovine 50 K array (Neogen Corporation) (52,260 SNP; 677 animals) and BovineHD BeadChips (Illumina Inc.) (606,006 SNP; 64 animals) SNP panels by *Neogen* (*GeneSeek*, A *Neogen* Company). These individuals were chosen to be genotyped based on pedigree‐based relatedness (genetic connectedness) aiming to capture as much genetic diversity as possible in animals with DNA samples, coming from nine representative NSIP Rambouillet flocks. Connectedness values were derived from prediction error co‐variances among EBV (Kuehn et al., 2007, 2008; Lewis et al., 2005) for preadjusted PWT. In these analyses, an animal model was fitted with CG as fixed, and additive and residual effects as random. As an additional criterion, animals with phenotypic information, either on themselves or on their progeny, for most traits analysed were prioritized for genotyping.

Approximately 35 K (35,105) autosomal SNP were in common between the two panels. The QC for the genotypic data was done using the plink 1.9 software (Purcell et al., 2007), with SNP markers with MAF < 0.05, call rate < 0.90, extreme departure from Hardy–Weinberg equilibrium (*P* < 10^−8^), located on non‐autosomal chromosomes, or a duplicate on the array removed. Samples with call rate < 0.90 were also removed. A total of 32,584 SNP and 722 samples remained for further analyses. The average distance between adjacent markers was 75.433 kb, with standard deviation, minimum, median, and maximum equal to 67.275 kb, 0.002 kb, 59.492 kb, and 2307.471 kb, respectively, in the 32 K panel used for the analyses.

### 2.3 Haplotype construction

The SNP genotypes for all samples were phased using the fimpute v.3.0 software (Sargolzaei et al., 2014) to infer the parental inheritance (i.e., which allele came from which parent), before creating the haplotype blocks. LD haploblocks were constructed using the $r^{2}$ metric (Hill & Robertson, 1968) with the thresholds of 0.15, 0.35, 0.50, 0.65, and 0.80 and based on the Big‐LD approach (Kim et al., 2018). The “gpart” package (Kim et al., 2019) implemented in r (R Core Team, 2020) was used to build the haploblocks.

### 2.4 Genetic evaluation

#### 2.4.1 Pedigree‐based predictions

Three linear mixed models for the pedigree‐based BLUP (A‐BLUP) were used in this study, which are defined as follow:

(1) $$y_{1}=1^{’}\mu +\mathrm{Zu}+e,$$

(2) $$y_{2}=1^{\prime }\mu +\mathrm{Zu}+\mathrm{Wp}+e,$$

(3) $$y_{3}=1^{\prime }\mu +\mathrm{Zu}+Z_{2}m+\mathrm{Sq}+e,$$

where the model (1) is an additive genetic model with $y_{1}$ representing a vector of single corrected phenotypic records, μ is the overall mean, u is the random direct additive genetic effect, and e is the random residual. The model (2) is a repeatability model, in which p is the random permanent environment effect, $y_{2}$ contains the repeated corrected phenotypic records, and the other vectors are the same as in model (1). The model (3) also includes the random maternal additive genetic and maternal permanent environment effects, m and q, respectively. The $1^{\prime }$ is a vector of ones used to calculate the overall mean and Z, W, $Z_{2}$, and S are the incidence matrices that relates the corrected phenotypic records to the random direct additive, permanent environment, maternal additive, and maternal permanent environment effects, respectively. The random effects for the above models were assumed to be normally distributed with (co)variance structures as follows:

(4) $$\text{Model}\;\left(1\right):\mathrm{Var}\left[\begin{array}{c} u\\ e \end{array}\right]=\left[\begin{array}{cc} A\sigma_{u}^{2} & 0\\ 0 & I\sigma_{e}^{2} \end{array}\right],$$

(5) $$\text{Model}\;\left(2\right):\mathrm{Var}\left[\begin{array}{c} u\\ p\\ e \end{array}\right]=\left[\begin{array}{ccc} A\sigma_{u}^{2} & 0 & 0\\ 0 & I\sigma_{p}^{2} & 0\\ 0 & 0 & I\sigma_{e}^{2} \end{array}\right],$$

(6) $$\text{Model}\;\left(3\right):\mathrm{Var}\left[\begin{array}{c} \begin{array}{c} u\\ m\\ q \end{array}\\ e \end{array}\right]=\left[\begin{array}{cccc} A\sigma_{u}^{2} & 0 & 0 & 0\\ 0 & A\sigma_{m}^{2} & 0 & 0\\ 0 & 0 & I\sigma_{q}^{2} & 0\\ 0 & 0 & 0 & I\sigma_{e}^{2} \end{array}\right],$$

where $\sigma_{u}^{2}$, $\sigma_{p}^{2}$, $\sigma_{m}^{2}$, $\sigma_{q}^{2}$, and$\;\sigma_{e}^{2}$ are the additive genetic, permanent environment, maternal genetic, maternal permanent environment, and residual variances, respectively, and model (1) was used to make the EBV prediction for the YFD, model (2) for NLB, and model (3) for BWT, PWT, YWT, and YGFW.

The blupf90 software (Misztal et al., 2018) was used to predict the EBV assuming the variance components were known (Table 2). To be consistent with the national genetic evaluation underway in Rambouillet sheep, the models fitted and the variance components used to predict the EBV were provided by NSIP.

**TABLE 2:** Variance components and genetic parameters used to predict the estimated breeding values for birth weight (BWT), postweaning body weight (PWT), yearling body weight (YWT), yearling fibre diameter (YFD), yearling greasy fleece weight (YGFW), and number of lambs born (NLB) in Rambouillet sheep

Parameter^a^	BWT	PWT	YWT	YFD	YGFW	NLB
$\sigma_{u}^{2}$	0.085	3.211	15.402	1.311	0.122	0.025
$\sigma_{p}^{2}$	–	–	–	–	–	0.009
$\sigma_{m}^{2}$	0.091	1.926	1.777	–	0.013	–
$\sigma_{q}^{2}$	0.061	1.926	1.777	–	0.013	–
$\sigma_{e}^{2}$	0.372	25.046	40.283	0.989	0.181	0.250
$\sigma_{p}^{2}$	0.610	32.110	59.240	2.300	0.330	0.284
$h^{2}$	0.140	0.100	0.260	0.570	0.370	0.090
$p^{2}$	–	–	–	–	–	0.030
$h_{m}^{2}$	0.150	0.060	0.030	–	0.040	–
$c^{2}$	0.100	0.060	0.030	–	0.040	–

^a^ $\sigma_{u}^{2}$ = additive genetic variance, $\sigma_{p}^{2}$ = permanent environment variance associated with repeated records, $\sigma_{m}^{2}$ = maternal additive genetic variance, $\sigma_{q}^{2}$ = maternal permanent environment variance, $\sigma_{e}^{2}$ = residual variance, $\sigma_{p}^{2}$ = phenotypic variance, $h^{2}$ = heritability for the direct additive genetic effect, $p^{2}$ = repeatability,${\;h}_{m}^{2}$ = heritability for the maternal additive genetic effect, $c^{2}$ = fraction of the phenotypic variance explained by the maternal permanent environment effect.

#### 2.4.2 Single‐step genomic BLUP using SNP

The corrected phenotypes, models, and variance components used to predict the GEBV under the single‐step genomic BLUP using SNP (H‐BLUP) approach were similar to the ones used in A‐BLUP, except for the inclusion of genomic relationships from the genotyped individuals. In the assumptions of the H‐BLUP, the y vector had corrected phenotypes for genotyped and non‐genotyped animals and $u\sim N\left(0,H\sigma_{u}^{2}\right)$. H is a relationship matrix that combines the pedigree and the genomic relationship matrices (Legarra et al., 2009), with its inverse computed as follows (Aguilar et al., 2010):

(7) $$H^{-1}=A^{-1}+\left[\begin{array}{cc} 0 & 0\\ 0 & \tau {\left(\alpha G+\beta A_{22}\right)}^{-1}-\omega A_{22}^{-1} \end{array}\right],$$

where $A^{-1}$ is the inverse of the pedigree relationship matrix, $A_{22}$ and $A_{22}^{-1}$ are the pedigree relationship matrix for the genotyped animals and its inverse, respectively, and G is the genomic relationship matrix calculated as proposed by Vanraden (2008):

(8) $$G=\frac{{\mathrm{MM}}^{\prime }}{2\sum p_{i}\left(1-p_{i}\right)},$$

where M has the dimension of *n* genotyped animals by *m* SNP markers (coded as 0, 1, or 2 for the absence, presence of one copy, or presence of two copies of the reference allele, respectively) and is centred based on twice of the allelic frequencies ($p_{i}$; $1-p_{i}$). The pregsf90 software (Misztal et al., 2018) was used to create the $H^{-1}$ matrix including the pedigree information, with τ and ω parameters assumed as the default values (1.0). Different values of α and β were used to evaluate the impact of increasing the proportion of $A_{22}$ on G, with α = 0.95 and 0.50, and thereby β = 0.05 and 0.50, respectively, in which the former is the default value in the pregsf90 software and the later was suggested as the choice in Terminal Sire sheep populations (McMillan & Swan, 2017).

#### 2.4.3 Single‐step genomic BLUP using haplotypes

The model and assumptions used in the single‐step genomic BLUP including haplotypes (HAP‐BLUP) approach were similar to those described for H‐BLUP. However, the G used in the ${\left(\alpha G+\beta A_{22}\right)}^{-1}$ component was constructed using non‐LD‐clustered SNP (NCSNP) and ps‐SNP. A ps‐SNP corresponds to one of the unique haplotype alleles present within a haploblock, coded as 0, 1, or 2 to account for the number of copies of the reference haplotype allele, similar to Teissier et al. (2020) and Araujo et al. (2021). As a haploblock can be multi‐allelic, several ps‐SNP can be created from a haploblock. The ps‐SNP were subjected to the same QC criteria as the SNP before their use for genomic prediction. The number of NCSNP plus ps‐SNP before QC ranged between 33,922 and 44,695 with the LD thresholds of 0.80 and 0.15, respectively, while the number of NCSNP plus ps‐SNP after QC (markers used in the haplotype predictions) ranged from 32,649 to 39,787 with the same LD thresholds (Appendix S1: Table S1). All the scenarios regarding the different combinations of α and β parameters described for H‐GBLUP were also tested in the HAP‐BLUP method.

### 2.5 Comparing genetic and genomic predictions

The whole (complete) and partial datasets used to compare the genetic and genomic predictions (Legarra & Reverter, 2018) for each trait (Table 1) were defined separately based on time thresholds considering the birth date of the genotyped animals as the reference. The whole datasets included all corrected phenotypic records and genotyped individuals with corrected phenotypes on itself or on its progeny. As the number of genotyped individuals was small, the division into partial datasets considered the following two criteria: (1) at least 100 genotyped individuals with average EBV accuracy higher than 0.50 as focal individuals (selection candidates with masked corrected phenotypes on itself and on its progeny) were kept; and (2) at least 20% of the genotyped individuals as focal individuals were kept. Initially, as the genotyped animals were born between 2009 and 2017, all individuals that were born in 2017 were elected to be focal individuals. However, with this approach it was not possible to keep at least 100 genotyped individuals in the focal set for all traits. Ultimately, different dates were used to divide the whole data in the partial datasets (April 21, 2016 for BWT and PWT, April 13, 2016 for YWT, April 26, 2016 for YFD, April 20, 2016 for YGDW, and 04/06/2016 for NLB).

The number of flocks included in the whole dataset for BWT, PWT, YWT, YFD, YGFW, and NLB were 15, 23, 17, 19, 19, 15, respectively, and in the partial datasets were 13, 13, 11, 12, 12, 12, respectively. We used the variance of the difference in unit effects (VED) to evaluate the connectedness between the genotyped samples considered as training and validation (focal) in the whole and partial datasets. The VED is based on the variance of unit effects, which approximates the mean prediction error variance (PEV) over unities (Kennedy & Trus, 1993), and, as in the PEV, the lower the value the more connected are the units under consideration. The “GCA” r package (Yu & Morota, 2021) was used to calculate the VED, in which the choice for the VED statistic was done due to calculating the connectedness in a single‐step and being less computationally expensive than PEV of the difference. The VED for the individuals used to evaluate the genomic predictions for BWT, PWT, YWT, YFD, YGFW, and NLB were 0.046, 0.059, 0.031, 0.008, 0.017, and 0.282, respectively, suggesting sufficient connectedness as the values were low, especially for BWT, PWT, YWT, YFD, and YGFW.

The performance of genetic and genomic predictions was evaluated using the linear regression (LR) method as described by Legarra and Reverter (2018). The LR method provides a series of statistics derived from the comparison of genetic evaluations using the whole and partial datasets, resulting in easy‐to‐use methods to evaluate the reliability of the predictions (Legarra & Reverter, 2018). The LR statistics obtained were:

(9) $$\text{Accuracy}=\sqrt{\frac{\mathrm{cov}\left(\left(G\right)\mathrm{EB}V_{W},\left(G\right)\mathrm{EB}V_{P}\right)}{\left(1-\bar{F}\right)\sigma_{u}^{2}}},$$

(10) $$\text{Bias}=\mathrm{ave}\left(\hat{u_{\left(G\right)\mathrm{EB}V_{P}}}\right)-\mathrm{ave}\left(\hat{u_{\left(G\right)\mathrm{EB}V_{W}}}\right),$$

(11) $$\text{Dispersion}=\left(\frac{\mathrm{cov}\left(\left(G\right)\mathrm{EB}V_{W},\left(G\right)\mathrm{EB}V_{P}\right)}{\mathrm{var}\left(\left(G\right)\mathrm{EB}V_{P}\right)}\right)-1,$$

where $\mathrm{cov}\left(\left(G\right)\mathrm{EB}V_{W},\left(G\right)\mathrm{EB}V_{P}\right)$ is the covariance between the GEBV or EBV in whole ($\left(G\right)\mathrm{EB}V_{W}$) and partial ($\left(G\right)\mathrm{EB}V_{P}$) datasets, $\bar{F}$ is the average inbreeding, $\mathrm{ave}\left(\right)$ represent the arithmetic average function, $\hat{u_{\left(G\right)\mathrm{EB}V_{W}}}$ and $\hat{u_{\left(G\right)\mathrm{EB}V_{P}}}$ are the predicted GEBV or EBV in the whole and partial datasets, respectively, and $\mathrm{var}\left(\left(G\right)\mathrm{EB}V_{P}\right)$ is the variance of the GEBV or EBV. The other components were previously described.

In addition to the LR statistics, the individual theoretical accuracies (TA) were calculated for the focal individuals according to Van Vleck (1993):

(12) $$\mathrm{TA}=\sqrt{1-\frac{{{\mathrm{se}}_{i}}^{2}}{\left(1+f_{i}\right)\sigma_{u}^{2}}},$$

where ${{\mathrm{se}}_{i}}^{2}$ is the square of the GEBV or EBV standard error for the individual *i*, $f_{i}$ is the inbreeding coefficient for the individual *i*, and the other variables were previously described. The TA was calculated for both whole and partial datasets but presented only for the whole dataset to highlight the overall increase in the TA comparing GEBV and EBV considering all phenotypes available.

### 2.6 Evaluated scenarios

The scenarios consisted of combinations of (1) A‐BLUP, (2) H‐BLUP with α = 0.95 and 0.50 to construct G, and (3) HAP‐BLUP using ps‐SNP from different LD thresholds (0.15, 0.35, 0.50, 0.65, and 0.80) also with α = 0.95 and 0.50 to construct G. In total, 13 scenarios were evaluated for each of the six traits, resulting in 78 analyses.

## 3 RESULTS

### 3.1 Genomic prediction accuracies

**FIGURE 1:**
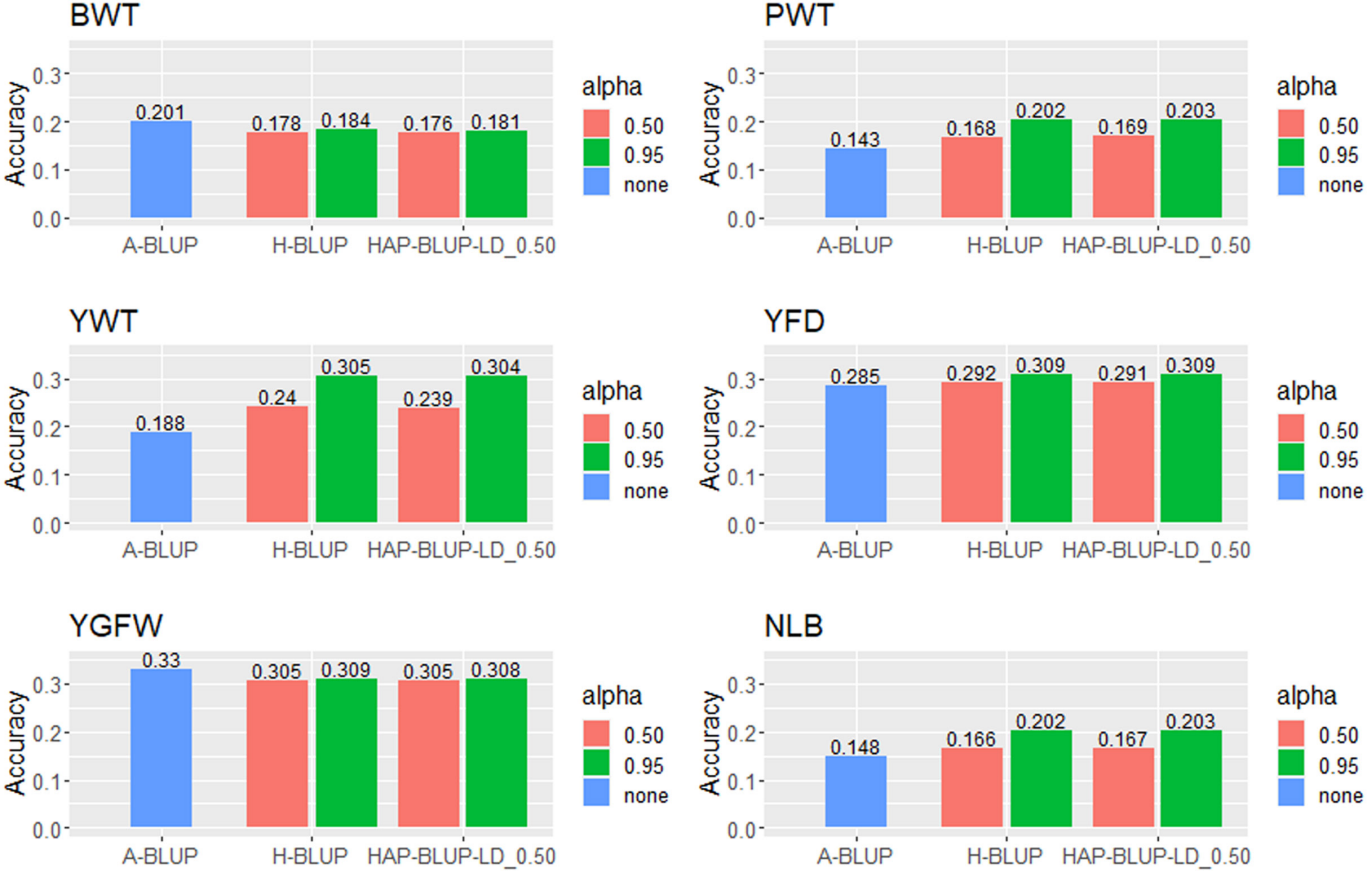
Prediction accuracies for birth weight (BWT), postweaning weight (PWT), yearling weight (YWT), yearling fibre diameter (YFD), yearling greasy fleece weight (YGFW), and number of lambs born (NLB) in Rambouillet sheep using pedigree BLUP (A‐BLUP), SNP‐based single‐step GBLUP (H‐BLUP), and haplotype‐based single‐step GBLUP fitting haplotypes from blocks with LD threshold of 0.50 (HAP‐BLUP‐LD_0.50). Different α values (0.50 or 0.95) were used to create the genomic relationship matrices [Colour figure can be viewed at wileyonlinelibrary.com]

All the detailed results for the genetic and genomic predictions using the 32 K panel including or not the haplotypes are present in the Appendix S2: Tables S1–S6. The prediction accuracies for body weight, wool, and NLB traits ranged from 0.143 (A‐BLUP for PWT; Appendix S2: Table S2) to 0.330 (A‐BLUP for YGFW; Appendix S2: Table S5). The lowest and highest prediction accuracies were observed for NLB and wool (both YFD and YGFW) traits, respectively. Similar prediction accuracies were observed for the HAP‐BLUP across different LD thresholds, regardless of the α value and trait evaluated. We, therefore, only presented the results for the HAP‐BLUP considering the LD threshold of 0.50 (HAP‐BLUP‐LD_0.50) to compare the predictions between pedigree, SNP, and haplotype‐based methods for all traits (Figure 1).

Using genomic information provided similar or higher GEBV prediction accuracies compared to EBV, except for BWT and YGFW. An increase of ~41% (~0.06), ~62% (~0.12), ~8% (~0.02), and ~37% (~0.05) in the GEBV prediction accuracies was observed for the PWT, YWT, YFD, and NLB, respectively, when using α equal to 0.95. Using an α of 0.50 generally resulted in half of the increase in the prediction accuracy compared to 0.95. No gains in GEBV accuracy were observed for BWT and YGFW by using genomic information. The increase in the accuracy using the SNP‐ and haplotype‐based models were similar, with differences smaller than 1% for all traits.

### 3.2 Bias

**FIGURE 2:**
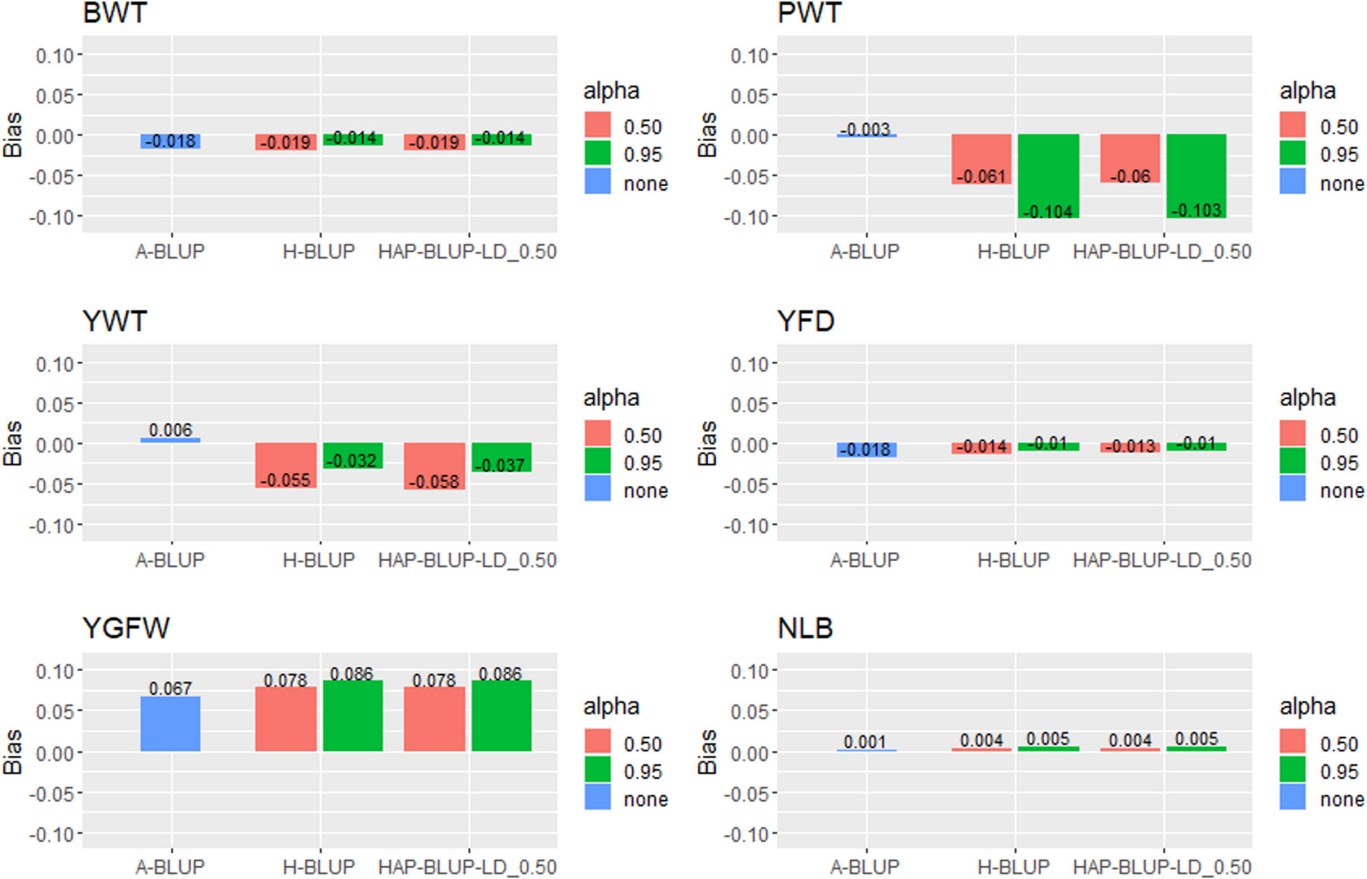
Prediction bias for birth weight (BWT), postweaning weight (PWT), yearling weight (YWT), yearling fibre diameter (YFD), yearling greasy fleece weight (YGFW), and number of lambs born (NLB) in Rambouillet sheep using pedigree BLUP (A‐BLUP), SNP‐based single‐step GBLUP (H‐BLUP), and haplotype‐based single‐step GBLUP fitting haplotypes from blocks with LD threshold of 0.50 (HAP‐BLUP‐LD_0.50). Different α values (0.50 or 0.95) were used to create the genomic relationship matrices [Colour figure can be viewed at wileyonlinelibrary.com]

The prediction bias ranged between −0.104 (H‐BLUP for PWT; Appendix S2: Table S2) and 0.087 (HAP‐BLUP using haplotypes from blocks with LD threshold of 0.15 and α of 0.95 for YGFW; Appendix S2: Table S5). Different from what was observed for the GEBV accuracies, the predictions for NLB were in general less biased than the other traits while those for PWT were the most biased. The prediction bias for the haplotype‐based methods was similar across LD thresholds (used to create the haploblocks) and, thus, only the HAP‐BLUP‐LD_0.50 were presented for comparison purposes (Figure 2).

Incorporating genomic information in the analyses resulted in similar or more bias when compared to the pedigree‐based prediction. Alpha equal to 0.95 tended to reduce the bias for BWT and YWT, while the opposite was observed for the other traits (i.e., using α = 0.50 reduced the prediction bias for the other traits).

### 3.3 Dispersion

**FIGURE 3:**
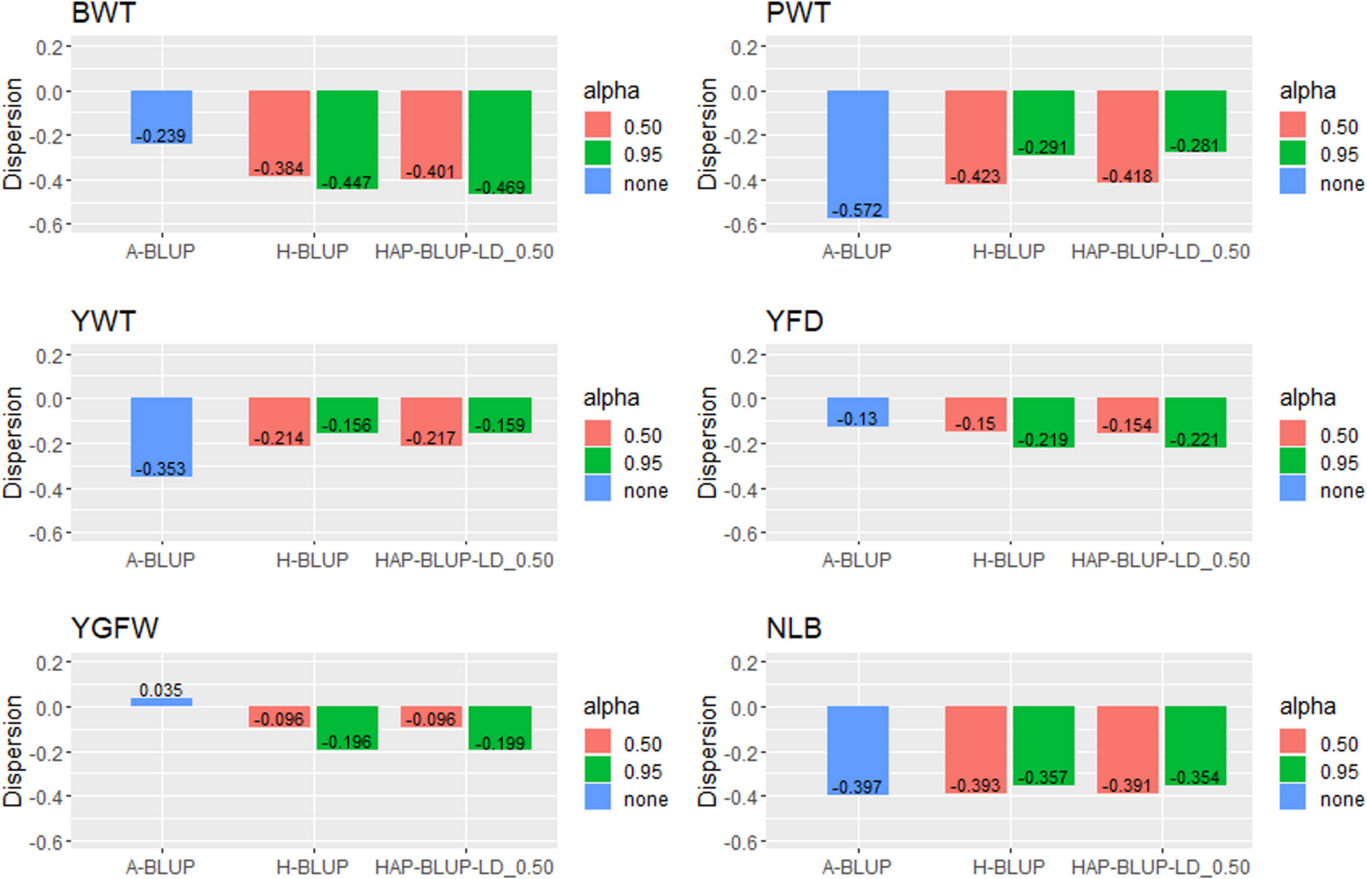
Dispersion of the genomic‐enhanced breeding values (GEBV) for birth weight (BWT), postweaning weight (PWT), yearling weight (YWT), yearling fibre diameter (YFD), yearling greasy fleece weight (YGFW), and number of lambs born (NLB) in Rambouillet sheep using pedigree BLUP (A‐BLUP), SNP‐based single‐step GBLUP (H‐BLUP), and haplotype‐based single‐step GBLUP using haplotypes from blocks with LD threshold of 0.50 (HAP‐BLUP‐LD_0.50). Different α values (0.50 or 0.95) were used to create the genomic relationship matrices [Colour figure can be viewed at wileyonlinelibrary.com]

The GEBV dispersion ranged from −0.572 (A‐BLUP for PWT; Appendix S2: Table S2) to 0.035 (A‐BLUP for YGFW; Appendix S2: Table S5). The dispersion was closer to zero (expected value for this statistic under no dispersion) for YGFW while it was more distant from (and typically below) zero for BWT and PWT indicating GEBV were overestimated. GEBV predictions using haplotypes from blocks with different LD thresholds resulted in similar dispersion of the GEBV. Therefore, the HAP‐BLUP‐LD_0.50 scenario was also used to represent the haplotype‐based methods to compare with A‐BLUP and H‐BLUP (Figure 3).

A dispersion of −0.29, −0.16, and − 0.35 was observed for PWT, YWT, and NLB, respectively, using H‐BLUP and HAP‐BLUP‐LD_0.50 with α of 0.95. Those values were closer to zero than when using A‐BLUP (−0.57, −0.35, and −0.39, respectively), showing reduced dispersion for genomic‐based models. Pedigree‐based models presented similar or lower dispersion for BWT and wool traits. The dispersion with H‐BLUP and HAP‐BLUP‐LD_0.50 showed similar results regardless of the α values. Alpha equal to 0.95 tended to present better dispersion for PWT, YWT, and NLB compared to 0.50, while the opposite was observed for the other traits.

### 3.4 Theoretical accuracy

**FIGURE 4:**
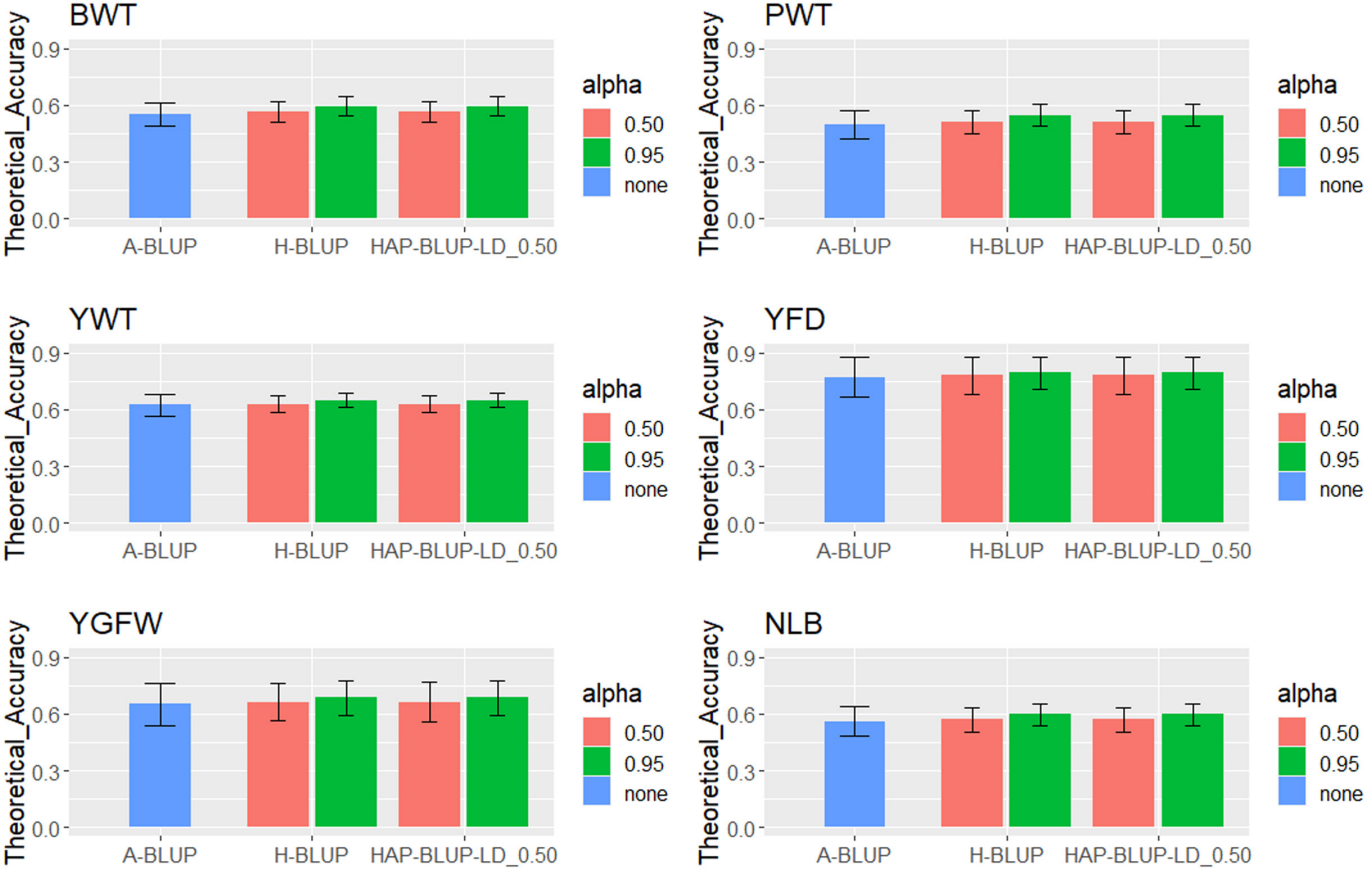
Mean theoretical accuracies for birth weight (BWT), postweaning weight (PWT), yearling weight (YWT), yearling fibre diameter (YFD), yearling greasy fleece weight (YGFW), and number of lambs born (NLB) in Rambouillet sheep using pedigree BLUP (A‐BLUP), SNP‐based single‐step GBLUP (H‐BLUP), and haplotype‐based single‐step GBLUP using haplotypes from blocks with linkage disequilibrium (LD) threshold of 0.50 (HAP‐BLUP‐LD_0.50). Different α values (0.50 or 0.95) were used to create the genomic relationship matrices [Colour figure can be viewed at wileyonlinelibrary.com]

The mean TA ranged from 0.499 (A‐BLUP for PWT; Appendix S2: Table S2) to 0.795 (HAP‐BLUP using haplotypes from blocks with LD threshold of 0.35 and alpha equal to 0.95 for YFD; Appendix S2: Table S4). Considering all traits, the mean TA was 0.631 (0.085) and TA values were higher for YFD and lower for PWT. Results from the haplotype‐based methods had similar mean TA regardless of the LD threshold used to construct the haploblocks for all traits. The HAP‐BLUP‐LD_0.50 was, therefore, again used to represent the HAP‐BLUP methods (Figure 4).

**FIGURE 5:**
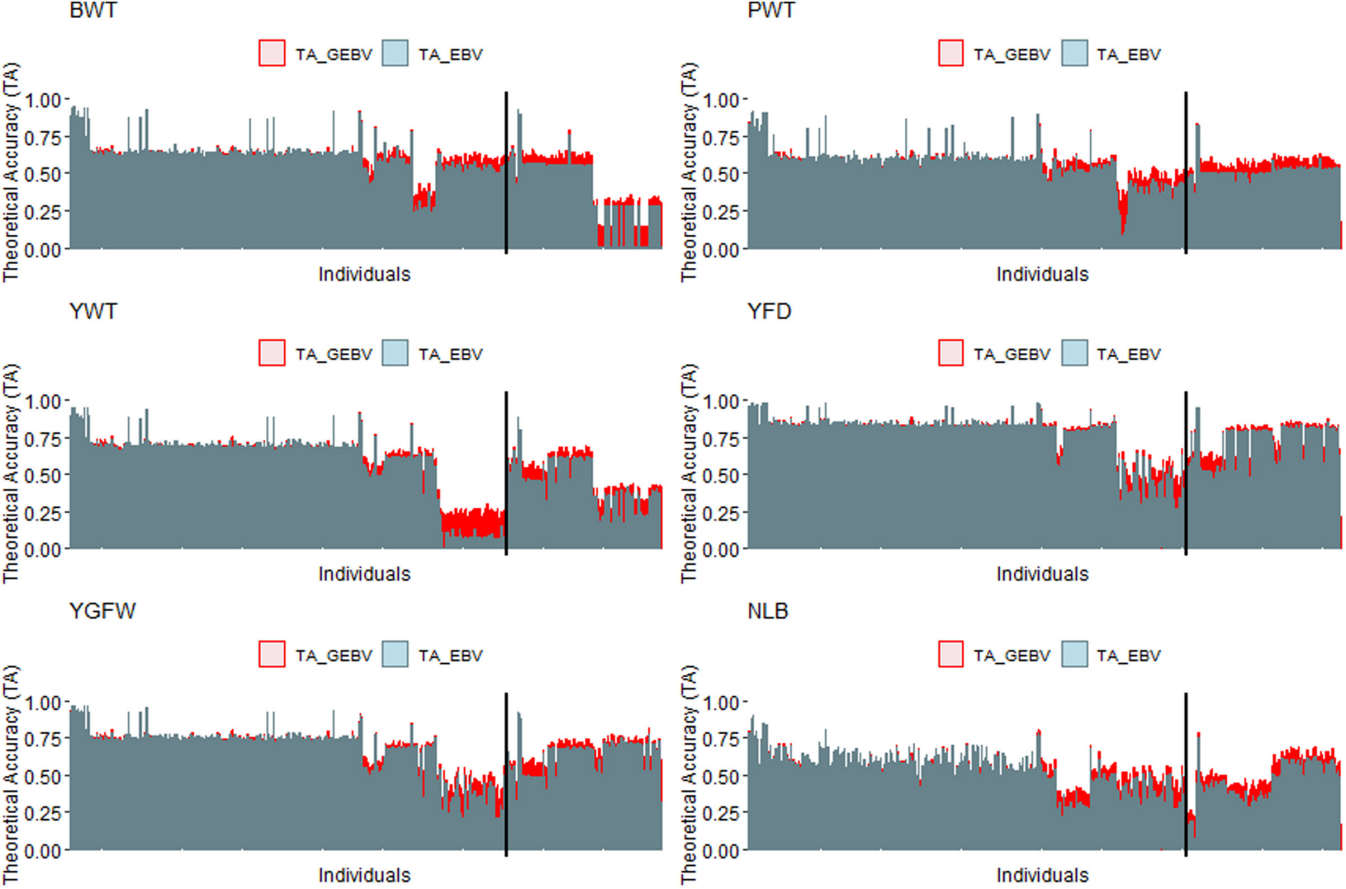
Theoretical accuracies for the genomic estimated breeding values using SNP (TA_GEBV) and estimated breeding values (TA_EBV) per genotyped individuals for birth weight (BWT), postweaning weight (PWT), yearling weight (YWT), yearling fibre diameter (YFD), yearling greasy fleece weight (YGFW), and number of lambs born (NLB). The TA_GEBV and TA_EBV were obtained using SNP in the single‐step GBLUP (H‐BLUP) with alpha equal to 0.95 and pedigree‐based BLUP (A‐BLUP), respectively. The individuals were sorted by birth date, so that the youngest individuals are in the right side of each plot. The black vertical line divides the training individuals in the partial datasets, with the focal animals on the right side of the line [Colour figure can be viewed at wileyonlinelibrary.com]

The genomic information tended to improve the mean TA for all traits, with increases up to ~7% (~0.04), ~9% (~0.05), ~4% (~0.03), ~3% (~0.02), ~5% (~0.03), and ~6% (~0.04) for BWT, PWT, YWT, YFD, YGFW, and NLB, respectively, using H‐BLUP and HAP‐BLUP‐LD_0.50 with α of 0.95. Negligible difference (less than 1%) was observed in the increase of the mean TA between H‐BLUP and HAP‐BLUP‐LD_0.50 with α of 0.95. Using αequal to 0.50 resulted in the smallest increase in the mean TA (less than 2%) with both SNP‐ and haplotype‐based methods for all traits. At the individual level, the TA using H‐BLUP with α of 0.95 were higher compared to A‐BLUP for the younger individuals and those with no phenotypic information (sires and dams with genotyped progeny) in the partial datasets for all traits (Figure 5).

Increase in the TA for ungenotyped close relatives (progeny or parents) of the genotyped individuals when using genomic information was also observed (Appendix S3: Figure S1), although the increases were smaller than those for the genotyped individuals. In the case of ungenotyped unrelated (animals with no pedigree links) individuals to the genotyped cohort, most of the TA with or without genomics were similar (Appendix S3: Figure S2); only few of these unrelated individuals benefited from the use of genomic information.

## 4 DISCUSSION

Genomic selection is the state‐of‐the‐art in modern sheep breeding programs. Here we present the first results of genomic predictions for body weight, wool, and reproductive traits in North American Rambouillet sheep. We performed single‐step genomic predictions fitting SNP or haplotypes to create the genomic relationship matrices used to compute GEBV. Despite the small dataset, promising results were obtained, which can be used as a starting point for the implementation of genomic selection in Rambouillet sheep as well as in other sheep populations.

### 4.1 Genetic and genomic prediction results

The accuracy of genomic predictions relies mainly on the trait heritability, LD between SNP and QTL (Meuwissen et al., 2001), population structure, and genetic diversity of the population (Daetwyler et al., 2012). For Rambouillet sheep, the prediction accuracies for the pedigree‐ and genomic‐based models followed the expected pattern in being higher for more heritable traits (Table 1; Figure 1). Correspondingly, the smallest (~0.02 in absolute value, ~8%) differences between the pedigree‐ and genomic‐based prediction accuracies were observed for YFD, which was the trait with the highest heritability (0.57), in comparison to NLB (~0.05 in absolute value, ~37%)—the trait with lowest heritability (0.09).

Despite the expectation of the theoretical accuracies of genomic predictions to be higher for traits with higher heritability (Meuwissen et al., 2001), benefits of using GEBV are expected to be higher for traits with low heritability, sex‐limited, hard‐to‐measure, and recorded late in life, especially in sheep (Brito, Clarke, et al., 2017; Brown et al., 2018). For more highly heritable and easily measured traits, given that the assumptions for the MME (Henderson, 1984) are met (e.g., polygenic architecture, deep and accurate pedigree data, large number of phenotypic records, no preselection), the EBV are expected to be BLUP and predict the unknown true breeding values well. In other words, the prediction accuracies using A‐BLUP for highly heritable traits where individuals and/or their progeny have phenotypes are already expected to be high.

As the genotyped cohort used in this study is the first official attempt to create a training population for genomic evaluations of Rambouillet sheep, the genotyped individuals included key ancestors and other selected animals with phenotypes or representative progeny with phenotypes for the traits evaluated. In this case, the EBV for those individuals were well estimated, especially for YFD and YGFW (higher heritabilities; Table 2), with small and negligible increase in the prediction accuracy for the genomic‐ compared to pedigree‐based models. For such highly heritable traits, using GEBV would still be more important to select breeding candidates at a younger age, that is, measured only at yearling age. The substantial increase in the prediction accuracy of PWT, YWT, and NLB (higher than ~37%) shows that greater genetic gains can be achieved for these traits by including genomic information, as the accuracy is one of the main components of the selection response (Falconer & Mackay, 1996).

The genomic prediction accuracies observed in our study were within the range for most of the economic traits in sheep, which is between 0.20 and 0.50 according to Brown et al. (2018), especially when using α of 0.95 to construct the **G** matrix. Oliveira et al. (2021) observed prediction accuracies for BWT ranging from 0.06 to 0.13 using H‐BLUP for Norwegian White and New Zealand Composite sheep populations. Unlike what was observed in the current study, Moghaddar et al. (2019) showed accuracies for genomic predictions ranging between 0.40 to 0.60 for PWT, 0.30 to 0.40 for yearling clean fleece weight, and 0.30 to 0.50 for YFD using BayesR and GBLUP for purebred Merino and crosses between Merino and Border Leicester. Genomic prediction accuracies of 0.24 and 0.28 were observed for YGFW using GBLUP and BayesR, respectively, and 0.31 and 0.35 for YFD for the same methods, respectively, in Merino and crossed Merino (Bolormaa, Brown, et al., 2017). For NLB, Bolormaa, Swan, et al. (2017) reported genomic prediction accuracies ranging from 0.15 to 0.56 considering different validation strategies and prediction based on GBLUP and BayesR under cross‐validation approaches for Merino sheep. Those differences in the genomic prediction accuracies for the same traits are also related to the statistical model and validation method used in the evaluations, beyond the other factors previously mentioned (e.g., heritability, population structure, and genetic diversity). Also, GEBV accuracies were not calculated in the same way across all the studies, but the LR method used in this study is currently considered as the gold‐standard approach.

The regression coefficient of the adjusted (or corrected) phenotypes or EBV on the GEBV is usually used to measure the “bias” of GEBV (Brown et al., 2018; Gao et al., 2012; Moghaddar et al., 2019; Oliveira et al., 2021). This measure was assessed as dispersion in our study, as it represents how the GEBV were inflated or deflated (over‐ or under‐estimated, respectively). Prediction bias, as a property of the method and the population under evaluation, is the expectation of the difference between average true and predicted breeding value; bias is zero under ideal conditions and can be approximated by the difference between the average (G)EBV in the whole and partial data sets (Legarra & Reverter, 2018). The observation that GEBV and EBV for most of the scenarios across traits were over‐estimated in the Rambouillet sheep was consistent with other studies (Brown et al., 2018; Moghaddar et al., 2019; Oliveira et al., 2021). Our conclusions regarding the benefit of including genomic information in prediction based on dispersion followed the same pattern observed for the GEBV prediction accuracies; this was likely because they are affected by similar factors.

Reports of prediction bias in sheep, as described by Legarra and Reverter (2018), are scarce and were found only in dairy sheep (Macedo et al., 2020, 2022). The same is true for TA, although this is an important metric when reporting breeding values back to producers. Brito, Clarke, et al. (2017) observed TA values ranging from 0.25 to 0.49 across a range of growth, carcass, and meat quality traits, which are smaller than the values observed in the current study. The substantial increase in the TA (Figure 4) especially for the young individuals using genomic information (Figure 5) is promising because these are the key individuals that need to be ranked for selection purposes.

Importantly, ungenotyped close relatives of the genotyped animals also had greater TA when incorporating genomic information. This suggests that genotyping strategies considering family information could also be used to improve the TA for ungenotyped individuals through genetic links. Similar to this study, Massender et al. (2022) also found that including genomic information provided higher TA for genotyped individuals compared to pedigree‐based predictions in dairy goats, with increases for ungenotyped animals with genotyped relatives. Regular and accurate pedigree collection is therefore encouraged to establish genetic links and evaluate which ungenotyped individuals could benefit more from having genomic information. An improved pedigree collection is also likely to increase the pedigree depth and quality, being important to control the genetic diversity of the population, which affects the long‐term responses of the breeding program (Stachowicz et al., 2018). The PCI is an important measure of pedigree quality and the average observed in this study (0.57) was higher than those for Canadian Suffolk (0.48) and for the major Canadian sheep breeds combined (0.50) reported by Stachowicz et al. (2018); nevertheless, the higher the PCI the better.

Selective genotyping can result in maximum genetic response (Boligon et al., 2012), which could explain the improvements in the prediction results for most of the traits analysed using genomic information. Such was the case even when the predictions were based on a small number of individuals (242 to 632 for NLB and BWT, respectively) genotyped using a moderate SNP density panel (~32 K SNP). Most of the prediction accuracy using genomic information is due to population structure, as described by Daetwyler et al. (2012). Those authors showed that up to 86% of the prediction accuracy can be achieved by using only one chromosome in a multibreed sheep population. Although one chromosome was enough to capture the population structure, it was unlikely to contain all the QTL affecting a trait (Daetwyler et al., 2012). Nevertheless, the recommendation is to increase the SNP panel density, through genotyping and imputation, for genomic predictions so that both population structure and LD between marker and QTL are fully explored (Daetwyler et al., 2012). Evaluating weighted single‐step genomic predictions and genome‐wide associations has also been encouraged (Wang et al., 2012) as there may be important genomic regions that explain more of the total additive genetic variance for the traits of interest.

Selective genotyping can increase bias in the variance component estimation and therefore is not recommended for the breeding programs that only use phenotypes and pedigree relationships to drive selection decisions (Wang et al., 2020). The potential bias from selective genotyping was a reason for using the variance components provided by the NSIP, derived using solely phenotypic and pedigree information. Using random selection to choose the samples to be genotyped, as well as increasing the training population size, is recommended to avoid bias in both variance component estimation and GEBV, as more biased predictions were observed using genomic information for most of the traits (Figure 2).

### 4.2 Using different alpha values to construct the genomic relationship matrices

In general, appropriate α and βparameters have more impact in GEBV bias reduction (Gao et al., 2012). Despite greater GEBV accuracies for a higher (0.95) α value, 0.50 is the choice to create **G** in single‐step evaluations for a range of carcass traits in terminal sire sheep breeds in Australia (McMillan & Swan, 2017). According to these authors, an α equal to 0.50 was chosen because (1) when increasing α, accuracies increased until reaching an asymptote at around 0.50, which was not the case in the current study; (2) the GEBV using α between 0.50 and 0.95 were highly correlated; and (3) less variation was observed in the GEBV of genotyped individuals without phenotypes with α equal to 0.50; and (4) with higher α values, GEBV bias (over‐prediction) increased.

In this study, using α of 0.50 showed only half of the increase in the accuracies compared to 0.95. No clear advantage in GEBV bias, dispersion, or TA was observed with one α value compared to the other. Therefore, we recommend α of 0.95 for single‐step genomic evaluations in U.S. Rambouillet sheep. However, it is important to highlight that our study included a smaller number of genotyped individuals compared to McMillan and Swan (2017) and we used the LR method (Legarra & Reverter, 2018) to derive the GEBV accuracies, bias, and dispersion; they instead used cross‐validation to test their predictions with random assignment of individuals to groups. Furthermore, it is recommended to evaluate the impact of different α and βin the creation of genomic relationships in multiple trait models in the future, as these parameters can also change for different traits (Gao et al., 2012; McMillan & Swan, 2017) and pedigree quality.

### 4.3 Haplotype‐based single‐step genomic predictions

The HAP‐BLUP‐LD_0.50 scenario was chosen to represent the haplotype‐based methods because the LD of 0.50 was the level most likely to estimate the recombination hotspots properly, which are the specific points in the genome with higher probability of recombination (Kim et al., 2018). The use of haplotype‐based methods did not improve the prediction results for any of the traits analysed (accuracies, bias, dispersion, and TA) compared to fitting SNP in a real (as opposed to simulated data) sheep dataset. This may have been the consequence of not enough changes in the genomic relationships to result in differences in the GEBV to be more accurate, as the correlations between the G matrices using only SNP or SNP plus haplotypes were high (~0.99). Haplotype‐based genomic predictions could outperform SNP‐based models in sheep datasets because the former can capture epistasis and these populations could have more complex interactions within haplotype blocks due to higher effective population size. Liang et al. (2020) showed that epistasis was the main reason for higher GEBV accuracies when using haplotypes instead of SNP in seven traits in humans, which is a highly genetically diverse population (Park, 2011). However, in this study, the small number of genotyped individuals (*n* = 722) as well as the density of the SNP panel used (~32 K SNP) could have affected both SNP and haplotype genomic predictions.

The algorithm to create the LD‐based haploblocks and the method to code the haplotypes during the creation of the relationship matrix could also have affected the prediction results. There are several algorithms to create LD‐haploblocks, such as MATILDE (Pattaro et al., 2008), confidence interval (Gabriel et al., 2002), four gamete test (Wang et al., 2002), solid spine (Barrett et al., 2005), MIG++ (Taliun et al., 2014), S‐MIG++ (Taliun et al., 2016), and Big‐LD (Kim et al., 2018). We have used the Big‐LD algorithm to construct the haploblocks because the LD blocks produced by this method agree better with the true recombination hotspots (determined experimentally in the major histocompatibility complex region from semen of north‐European British donors) and are more computationally efficient than the previously mentioned algorithms (Kim et al., 2018). However, as the haplotype diversity index and true discovery ratio of the recombination hotspots can be lower using Big‐LD (Kim et al., 2018), evaluating different algorithms to create the LD‐haplotype blocks is also recommended. New methods based on clustering algorithms (Won et al., 2020) and machine learning methods (Lim et al., 2022) have been recently proposed to create and select the best haplotypes to be used, respectively. These approaches should be evaluated in future studies with larger genomic datasets and populations with different genetic backgrounds.

The haplotypes can be multiallelic markers (Calus et al., 2008; Gabriel et al., 2002). However, we used the unique multiallelic haplotype alleles coded as ps‐SNP to perform genomic predictions under the ssGBLUP framework. The ps‐SNP derived from the LD‐haploblocks were then merged with the NCSNP to create the G matrix, similar to Araujo et al. (2021). This strategy enables fitting haplotypes to perform genomic predictions using software developed for fitting individual SNP (Teissier et al., 2020), including or excluding non‐genotyped individuals (ssGBLUP and GBLUP, respectively). Teissier et al. (2020) considered both NCSNP and unique multiallelic haplotype alleles as ps‐SNP and observed up to 22% increase in GEBV prediction accuracy when using different LD‐ or fixed‐SNP‐length‐based haploblocks for milk production traits in dairy goats using ssGBLUP. Milk production traits are known to be affected by a major gene (*DGAT1*). In general, GEBV prediction results for haplotype‐based methods are scarce in small ruminants and additional studies are needed.

The GVCHAP is a computing pipeline that allows multiallelic haplotypes to be used directly to create a genomic additive (and dominance) relationship matrix for both genomic prediction and variance component estimation using haplotypes or SNP (Prakapenka et al., 2020). GVCHAP is based in the multiallelic haplotype model proposed by Da (2015), which uses the quantitative genetic theory to derive a general multiallelic partition of genotypic values with factorization to define the genomic relationships. However, the GVCHAP is based on GREML and GBLUP and, thus, only considers genotyped individuals with phenotypes. Considering the different algorithms and methods to create haploblocks, code the haplotype alleles, and create the genomic relationship matrix including haplotypes, there are still further alternatives to evaluate the feasibility of including haplotypes in genomic predictions. Future studies in sheep could also consider the possibility of creating haplotypes based on functional information (e.g., gene regions) to perform haplotype predictions (Da, 2015; Prakapenka et al., 2020).

Despite the hypothesis that haplotypes could outperform SNP and provide high accuracies and lower bias in genomic predictions, recent results have shown that this does not usually happen. As summarized by Araujo et al. (2021), the benefits of using haplotype‐based methods for genomic prediction are equivocal most of the time. Significant improvements usually occur mainly in the evaluation of traits with major genes, as shown by Teissier et al. (2020). Nevertheless, as stated before, there are haplotype blocking and selection methods that should be further investigated. There are methods that might be able to better capture the haplotype variation and improve GEBV accuracy.

Marker density can also affect the accuracy of SNP phasing (Weng et al., 2014) and the precision in which the recombination hotspots are determined (Weng et al., 2019), which are the first steps for the haplotype prediction and the basis of the LD‐based haploblocks, respectively. In addition, epistasis, which is the component that might contribute the most to improvements in accuracy with haplotype predictions (Liang et al., 2020), is a complex effect and requires a substantial number of individuals and markers/bins to be properly estimated (Zhang et al., 2016).

Despite the fact that we genotyped individuals for SNP panel with a higher density (50 K and 600 K) than the one used in this research (35 K), genotype imputation prior to haplotype blocking and prediction can also be hampered by the number of reference individuals (Ventura et al., 2016). In early stages of this research, we imputed the 50 K panel from the overlapping 35 K SNP panel for those animals with HD genotypes. The results of genomic predictions using the imputed 50 K for both SNP and haplotype‐based methods (Appendix S4: Tables S1–S6) were similar to that using only the overlapping ~35 K SNPs, except for the YGFW (Appendix S4: Table S5). This likely happened because increasing the SNP density of only ~60 individuals by ~6 K SNP (imputed variants that passed a QC similar to that described in the section 2.2) to create the G matrix to be combined with A to create the $H^{-1}$ was not large enough to result in significant improvements in the genomic predictions. The imputation accuracy for SNP and individuals was ~80% and ~77%, respectively, which were below the 90% threshold suggested by Bolormaa et al. (2015).

Even though the accuracy of predictions for YGFW using haplotypes based on imputed 50 K panels was higher (up to ~0.09 compared to predictions using the 35 K panel), generally predictions using the imputed 50 K were more biased and the GEBV were more dispersed for all traits. Legarra and Reverter (2018) emphasized that accuracies should only be estimated and compared from empirically unbiased models. The results of the LR statistics using the imputed set of markers could have been affected by bias because of the imputation process due to limitations in the size of the reference population. Our results based on the 35 K SNP panel should therefore be used considering the Rambouillet sheep currently genotyped. Larger reference populations and denser SNP panels are recommended to evaluate genomic predictions using haplotypes in future sheep studies.

## 5 CONCLUSIONS

Using genomic information (SNP or haplotypes as pseudo‐SNP) provided similar or higher GEBV prediction and theoretical accuracies, and reduced the dispersion of the GEBV, for body weight, wool, and reproductive traits in Rambouillet sheep. However, there were no clear improvements in prediction bias compared to pedigree‐based predictions. Alpha value equal to 0.95 is recommended to weight the genomic relationships when modelling the covariances between individuals. The use of haplotypes showed no advantage compared to SNP at the current reference population size and SNP panel density used, regardless of the LD threshold used to create the haploblocks. SNP‐based genomic predictions are therefore recommended since they are easier to implement than those based on haplotypes. Efforts to increase the number of genotyped individuals are paramount to take full advantage of genomic information to accelerate genetic progress in the U.S. Rambouillet sheep breeding program.

## AUTHOR CONTRIBUTION

ACA, PLSC, RML, and LFB: conception of the work; RML and LFB: data acquisition; ACA: SNP and haplotype‐based single‐step genomic prediction analyses; ACA, RML, and LFB: results interpretation; ACA: drafting the manuscript; ACA, PLSC, HRO, RML, and LFB: critical revision of the manuscript; and, ACA, PLSC, HRO, RML, and LFB: final approval of the version to be published. All authors contributed to the article and approved the submitted version. All authors have read and agreed to the published version of the manuscript.

## FUNDING INFORMATION

This research was funded by the National Sheep Industry Improvement Center (NSIIC‐USDA), the American Rambouillet Sheep Breeders Association, and the American Sheep Industry Association Let us Grow program. The State University of Southwest Bahia and the Coordenação de Aperfeiçoamento de Pessoal de Nível Superior (CAPES, Brazil), Financial Code 001, provided the scholarship of the first author.

## CONFLICT OF INTEREST

The authors declare no conflict of interest.

## Supporting information


Appendix S1.

Appendix S2.

Appendix S3.

Appendix S4.

## Data Availability

The phenotypic, pedigree and genomic data used in this study are the property of the industry partner that contributed to the study and therefore are not readily available due to its commercial sensitivity. Requests to access the datasets should be directed to the National Sheep Improvement Program (NSIP). The computing pipelines used in this research are available by request to the corresponding authors.
