# Supplementary material for: SNP‐ and haplotype‐based single‐step genomic predictions for body weight, wool, and reproductive traits in North American Rambouillet sheep
Source: J Anim Breed Genet. 2022 Nov 21;140(2):216–34. doi: 10.1111/jbg.12748 (PMC10099590; doi:10.1111/jbg.12748)
Supplement: Supplementary file 1 — Appendix S1. [file JBG-140-216-s001.docx]

**SUPPLEMENTARY FILE 1**

**Supplementary File 1. Haplotype block structure considering different linkage disequilibrium (LD) thresholds**

The number of non-LD-clustered single nucleotide polymorphisms (NCSNP) increased from 17,872 to 32,085 with the linkage disequilibrium (LD) thresholds of 0.15 and 1.00, respectively, while the number of clustered single nucleotide polymorphisms (CSNP) decreased from 14,712 to 499 with these same LD thresholds, respectively (Supplementary File 1 Table 1). The number of haploblocks also decreased when increasing the LD thresholds, ranging between 222 and 6,840 with the LD thresholds of 1.00 and 0.15, respectively. The minimum number of SNP within haploblocks was constant and equal to two as previously defined, while the maximum number of SNP within haploblocks varied from 11 to 18 (LD thresholds of 1.00 and 0.35, respectively). The average number of SNP within haploblocks ranged from 2.15 to 2.25 (LD thresholds of 0.15 and 1.00, respectively), while the median number of SNP was equal to two SNP for all LD thresholds. The average haploblock size decreased from 0.020 to 0.008 Mb with the LD thresholds of 0.15 and 1.00, respectively. Surprisingly, the minimum block size was equal to 11 bp for the LD threshold of 1.00 and two bp for all haploblocks regardless of the LD threshold, while the maximum block size ranged from 0.049 to 0.164 Mb with the LD thresholds of 1.00 and 0.15, respectively. The minimum number of unique haplotype alleles per haploblock was equal to two for all LD thresholds, whereas the maximum ranged from 8 to 30 (LD thresholds of 1.00 and 0.65, respectively), the average ranged from 2.207 to 3.921 (LD thresholds of 1.00 and 0.15, respectively), and the median ranged from 2.00 (LD threshold of 1.00) to 4.00 (LD thresholds of 0.15, 0.35, and 0.50). The number of pseudo-SNP (ps-SNP; overall unique haplotype alleles) created was inversely proportional to the LD threshold and ranged between 490 and 26,823 for the LD thresholds of 1.00 and 0.15, respectively. Similar to the number of ps-SNP created, the number of NCSNP plus ps-SNP before and after quality control (QC) decreased when increasing the LD threshold. The number of NCSNP plus ps-SNP before QC ranged between 32,575 and 44,695 with the LD thresholds of 1.00 and 0.15, respectively, while the number of NCSNP plus ps-SNP after QC (markers used in the haplotype predictions) ranged from 32,649 to 39,787 based on these same LD thresholds.

**Supplementary File 1 Table 1.** Number of non-LD-clustered and clustered single nucleotide polymorphisms (NCSNP and CSNP, respectively); number of haploblocks (N_HB); minimum, maximum, average, and median for the number of SNP within haploblocks (Min_SNP, Max_SNP, Ave_SNP, and Median_SNP, respectively); average, minimum and maximum haploblock size (Ave_HBS, Min_HBS, and Max_HBS, respectively) ); minimum, maximum, average, and median for the number of alleles per haploblock (Min_ Hap_allele, Max_ Hap_allele, Ave_ Hap_allele, and Median_ Hap_allele, respectively); number of pseudo-SNP (ps-SNP) and NCSNP plus ps-SNP before and after quality control (NCSNP+ps-SNP_BQC and NCSNP+ps-SNP_AQC) obtained from haplotype analyses in Rambouillet sheep.

| Variable | Linkage Disequilibrium (LD) | | | | | |
| --- | --- | --- | --- | --- | --- | --- |
|  | 0.15 | 0.35 | 0.50 | 0.65 | 0.80 | 1.00^3^ |
| NCSNP | 17,872 | 21,976 | 24,871 | 27,385 | 29,521 | 32,085 |
| CSNP | 14,712 | 10,608 | 7,713 | 5,199 | 3,063 | 499 |
| N_HB | 6,840 | 4,907 | 3,513 | 2,358 | 1,388 | 222 |
| Min_SNP | 2 | 2 | 2 | 2 | 2 | 2 |
| Max_SNP | 13 | 18 | 13 | 15 | 15 | 11 |
| Ave_SNP  (SD)^1^ | 2.151 (0.550) | 2.162  (0.595) | 2.196 (0.669) | 2.205 (0.699) | 2.207 (0.783) | 2.248 (0.880) |
| Median_SNP | 2.000 | 2.000 | 2.000 | 2.000 | 2.000 | 2.000 |
| Ave_HBS (Mb)  (SD)^1^ | 0.020 (0.015) | 0.018 (0.014) | 0.017 (0.014) | 0.016 (0.013) | 0.014  (0.012) | 0.008 (0.009) |
| Min_HBS (bp) | 2 | 2 | 2 | 2 | 2 | 11 |
| Max_HBS (Mb) | 0.164 | 0.116 | 0.134 | 0.073 | 0.092 | 0.049 |
| Min_Hap_allele | 2 | 2 | 2 | 2 | 2 | 2 |
| Max_ Hap_allele | 26 | 26 | 28 | 30 | 13 | 8 |
| Ave_ Hap_allele | 3.921  (1.357) | 3.780 (1.259) | 3.692 (1.322) | 3.504 (1.367) | 3.171 (1.117) | 2.207 (0.774) |
| Median_ Hap_allele | 4.000 | 4.000 | 4.000 | 3.000 | 3.000 | 2.000 |
| ps-SNP^2^ | 26,823 | 18,547 | 12,971 | 8,263 | 4,401 | 490 |
| NCSNP+ps-SNP_BQC | 44,695 | 40,523 | 37,842 | 35,648 | 33,922 | 32,575 |
| NCSNP+ps-SNP_AQC | 39,787 | 36,679 | 34,781 | 33,473 | 32,649 | - |

^1^ Standard deviation. ^2^Pseudo-SNP are the unique haplotype alleles from the combination of phased SNP within haploblocks. ^3^Not used for the genomic predictions, only as reference to compare the haplotype block structure with the other LD thresholds.

Note that the maximum number of SNP in haploblocks is smaller for the LD threshold of 0.15 than for 0.35. This can happen because we used the Big-LD algorithm to create the haploblocks. The Big-LD method first gives priority to non-overlapping intervals with larger number of SNPs, then breaks the LD bins of markers using a marker density function to keep markers that are relatively close in physical proximity [please see Kim et al. (2018; <https://academic.oup.com/bioinformatics/article/34/3/388/4282661?login=true>) for more information]. In summary, after having long bins of markers with 0.15 threshold, the greedy algorithm described by the authors can break those intervals due to more overlapping and smaller density compared to 0.35 to keep the expected LD for the makers at the threshold level while minimizing the correlation among blocks.
