# Supplementary material for: SNP‐ and haplotype‐based single‐step genomic predictions for body weight, wool, and reproductive traits in North American Rambouillet sheep
Source: J Anim Breed Genet. 2022 Nov 21;140(2):216–34. doi: 10.1111/jbg.12748 (PMC10099590; doi:10.1111/jbg.12748)
Supplement: Supplementary file 2 — Appendix S2. [file JBG-140-216-s004.docx]

**SUPPLEMENTARY FILE 2**

**Supplementary File 2 Table 1.** Results of genetic and single-step genomic predictions for birth weight in Rambouillet sheep when using alpha equal to 0.95 or 0.50 to create the genomic relationship matrix.

| **Alpha** | **Method^1^** | **Accuracy** | **Bias** | **Dispersion** | **TA_mean^2^** | **TA_sd^3^** |
| --- | --- | --- | --- | --- | --- | --- |
| none | A-BLUP | 0.201 | -0.018 | -0.239 | 0.552 | 0.061 |
| 0.95 | H-BLUP | 0.184 | -0.014 | -0.447 | 0.593 | 0.051 |
| 0.95 | HAP-BLUP-LD_0.15 | 0.183 | -0.013 | -0.465 | 0.593 | 0.051 |
| 0.95 | HAP-BLUP-LD_0.35 | 0.181 | -0.014 | -0.471 | 0.593 | 0.051 |
| 0.95 | HAP-BLUP-LD_0.50 | 0.181 | -0.014 | -0.469 | 0.593 | 0.051 |
| 0.95 | HAP-BLUP-LD_0.65 | 0.184 | -0.014 | -0.448 | 0.593 | 0.051 |
| 0.95 | HAP-BLUP-LD_0.80 | 0.184 | -0.014 | -0.448 | 0.593 | 0.051 |
| 0.50 | H-BLUP | 0.178 | -0.019 | -0.384 | 0.564 | 0.053 |
| 0.50 | HAP-BLUP-LD_0.15 | 0.177 | -0.019 | -0.398 | 0.564 | 0.053 |
| 0.50 | HAP-BLUP-LD_0.35 | 0.175 | -0.019 | -0.405 | 0.564 | 0.053 |
| 0.50 | HAP-BLUP-LD_0.50 | 0.176 | -0.019 | -0.401 | 0.564 | 0.053 |
| 0.50 | HAP-BLUP-LD_0.65 | 0.178 | -0.019 | -0.384 | 0.564 | 0.053 |
| 0.50 | HAP-BLUP-LD_0.80 | 0.178 | -0.019 | -0.384 | 0.564 | 0.053 |

^1^ BLUP = Best Linear Unbiased Prediction; SNP = Single Nucleotide Polymorphisms; A-BLUP = pedigree-based BLUP; H-BLUP = SNP-based BLUP; HAP-BLUP-LD_0.15, HAP-BLUP-LD_0.35, HAP-BLUP-LD_0.50, HAP-BLUP-LD_0.65, HAP-BLUP-LD_0.80 = haplotype-based BLUP using non-clustered SNP and pseudo-SNP from haploblocks with linkage disequilibrium thresholds of 0.15, 0.35, 0.50, 0.65, and 0.80, respectively. ^2^ Mean theoretical accuracy. ^3^ Standard deviation for the mean theoretical accuracy.

**Supplementary File 2 Table 2.** Results of genetic and single-step genomic predictions for post weaning weight in Rambouillet sheep using alpha equal to 0.95 or 0.50 to create the genomic relationship matrix.

| **Alpha** | **Method^1^** | **Accuracy** | **Bias** | **Dispersion** | **TA_mean^2^** | **TA_sd^3^** |
| --- | --- | --- | --- | --- | --- | --- |
| none | A-BLUP | 0.143 | -0.003 | -0.572 | 0.499 | 0.073 |
| 0.95 | H-BLUP | 0.202 | -0.104 | -0.291 | 0.546 | 0.058 |
| 0.95 | HAP-BLUP-LD_0.15 | 0.203 | -0.103 | -0.274 | 0.546 | 0.058 |
| 0.95 | HAP-BLUP-LD_0.35 | 0.202 | -0.103 | -0.286 | 0.546 | 0.058 |
| 0.95 | HAP-BLUP-LD_0.50 | 0.203 | -0.103 | -0.281 | 0.546 | 0.058 |
| 0.95 | HAP-BLUP-LD_0.65 | 0.202 | -0.101 | -0.287 | 0.546 | 0.058 |
| 0.95 | HAP-BLUP-LD_0.80 | 0.202 | -0.102 | -0.285 | 0.546 | 0.058 |
| 0.50 | H-BLUP | 0.168 | -0.061 | -0.423 | 0.513 | 0.062 |
| 0.50 | HAP-BLUP-LD_0.15 | 0.170 | -0.061 | -0.413 | 0.513 | 0.062 |
| 0.50 | HAP-BLUP-LD_0.35 | 0.169 | -0.061 | -0.421 | 0.513 | 0.062 |
| 0.50 | HAP-BLUP-LD_0.50 | 0.169 | -0.060 | -0.418 | 0.513 | 0.062 |
| 0.50 | HAP-BLUP-LD_0.65 | 0.169 | -0.060 | -0.421 | 0.513 | 0.062 |
| 0.50 | HAP-BLUP-LD_0.80 | 0.169 | -0.060 | -0.420 | 0.513 | 0.062 |

^1^ BLUP = Best Linear Unbiased Prediction; SNP = Single Nucleotide Polymorphisms; A-BLUP = pedigree-based BLUP; H-BLUP = SNP-based BLUP; HAP-BLUP-LD_0.15, HAP-BLUP-LD_0.35, HAP-BLUP-LD_0.50, HAP-BLUP-LD_0.65, HAP-BLUP-LD_0.80 = haplotype-based BLUP using non-clustered SNPs and pseudo-SNPs from haploblocks with linkage disequilibrium thresholds of 0.15, 0.35, 0.50, 0.65, and 0.80, respectively. ^2^ Mean theoretical accuracy. ^3^ Standard deviation for the mean theoretical accuracy.

**Supplementary File 2 Table 3.** Results of genetic and single-step genomic predictions for yearling weight in Rambouillet sheep using alpha equal to 0.95 or 0.50 to create the genomic relationship matrix.

| **Alpha** | **Method^1^** | **Accuracy** | **Bias** | **Dispersion** | **TA_mean^2^** | **TA_sd^3^** |
| --- | --- | --- | --- | --- | --- | --- |
| none | A-BLUP | 0.188 | 0.006 | -0.353 | 0.624 | 0.055 |
| 0.95 | H-BLUP | 0.305 | -0.032 | -0.156 | 0.650 | 0.04 |
| 0.95 | HAP-BLUP-LD_0.15 | 0.307 | -0.043 | -0.154 | 0.650 | 0.04 |
| 0.95 | HAP-BLUP-LD_0.35 | 0.307 | -0.037 | -0.154 | 0.650 | 0.04 |
| 0.95 | HAP-BLUP-LD_0.50 | 0.304 | -0.037 | -0.159 | 0.650 | 0.04 |
| 0.95 | HAP-BLUP-LD_0.65 | 0.304 | -0.031 | -0.158 | 0.650 | 0.04 |
| 0.95 | HAP-BLUP-LD_0.80 | 0.304 | -0.031 | -0.154 | 0.650 | 0.04 |
| 0.50 | H-BLUP | 0.240 | -0.055 | -0.214 | 0.630 | 0.043 |
| 0.50 | HAP-BLUP-LD_0.15 | 0.241 | -0.061 | -0.213 | 0.630 | 0.043 |
| 0.50 | HAP-BLUP-LD_0.35 | 0.241 | -0.058 | -0.213 | 0.630 | 0.043 |
| 0.50 | HAP-BLUP-LD_0.50 | 0.239 | -0.058 | -0.217 | 0.630 | 0.043 |
| 0.50 | HAP-BLUP-LD_0.65 | 0.240 | -0.055 | -0.216 | 0.630 | 0.043 |
| 0.50 | HAP-BLUP-LD_0.80 | 0.240 | -0.054 | -0.213 | 0.630 | 0.043 |

^1^ BLUP = Best Linear Unbiased Prediction; SNP = Single Nucleotide Polymorphisms; A-BLUP = pedigree-based BLUP; H-BLUP = SNP-based BLUP; HAP-BLUP-LD_0.15, HAP-BLUP-LD_0.35, HAP-BLUP-LD_0.50, HAP-BLUP-LD_0.65, HAP-BLUP-LD_0.80 = haplotype-based BLUP using non-clustered SNPs and pseudo-SNPs from haploblocks with linkage disequilibrium thresholds of 0.15, 0.35, 0.50, 0.65, and 0.80, respectively. ^2^ Mean theoretical accuracy. ^3^ Standard deviation for the mean theoretical accuracy.

**Supplementary File 2 Table 4.** Results of genetic and single-step genomic predictions for yearling fiber diameter in Rambouillet sheep using alpha equal to 0.95 or 0.50 to create the genomic relationship matrix.

| **Alpha** | **Method^1^** | **Accuracy** | **Bias** | **Dispersion** | **TA_mean^2^** | **TA_sd^3^** |
| --- | --- | --- | --- | --- | --- | --- |
| none | A-BLUP | 0.285 | -0.018 | -0.130 | 0.771 | 0.105 |
| 0.95 | H-BLUP | 0.309 | -0.010 | -0.219 | 0.795 | 0.085 |
| 0.95 | HAP-BLUP-LD_0.15 | 0.312 | -0.008 | -0.230 | 0.795 | 0.085 |
| 0.95 | HAP-BLUP-LD_0.35 | 0.310 | -0.008 | -0.217 | 0.795 | 0.085 |
| 0.95 | HAP-BLUP-LD_0.50 | 0.309 | -0.010 | -0.221 | 0.795 | 0.085 |
| 0.95 | HAP-BLUP-LD_0.65 | 0.311 | -0.009 | -0.219 | 0.795 | 0.085 |
| 0.95 | HAP-BLUP-LD_0.80 | 0.310 | -0.010 | -0.219 | 0.795 | 0.085 |
| 0.50 | H-BLUP | 0.292 | -0.014 | -0.150 | 0.780 | 0.097 |
| 0.50 | HAP-BLUP-LD_0.15 | 0.293 | -0.013 | -0.159 | 0.780 | 0.097 |
| 0.50 | HAP-BLUP-LD_0.35 | 0.292 | -0.013 | -0.153 | 0.780 | 0.097 |
| 0.50 | HAP-BLUP-LD_0.50 | 0.291 | -0.013 | -0.154 | 0.780 | 0.097 |
| 0.50 | HAP-BLUP-LD_0.65 | 0.292 | -0.013 | -0.152 | 0.780 | 0.097 |
| 0.50 | HAP-BLUP-LD_0.80 | 0.292 | -0.014 | -0.152 | 0.780 | 0.097 |

^1^ BLUP = Best Linear Unbiased Prediction; SNP = Single Nucleotide Polymorphisms; A-BLUP = pedigree-based BLUP; H-BLUP = SNP-based BLUP; HAP-BLUP-LD_0.15, HAP-BLUP-LD_0.35, HAP-BLUP-LD_0.50, HAP-BLUP-LD_0.65, HAP-BLUP-LD_0.80 = haplotype-based BLUP using non-clustered SNPs and pseudo-SNPs from haploblocks with linkage disequilibrium thresholds of 0.15, 0.35, 0.50, 0.65, and 0.80, respectively. ^2^ Mean theoretical accuracy. ^3^ Standard deviation for the mean theoretical accuracy.

**Supplementary File 2 Table 5.** Results of genetic and single-step genomic predictions for yearling greasy fleece weight in Rambouillet sheep using alpha equal to 0.95 or 0.50 to create the genomic relationship matrix.

| **Alpha** | **Method^1^** | **Accuracy** | **Bias** | **Dispersion** | **TA_mean^2^** | **TA_sd^3^** |
| --- | --- | --- | --- | --- | --- | --- |
| none | A-BLUP | 0.330 | 0.067 | 0.035 | 0.653 | 0.112 |
| 0.95 | H-BLUP | 0.309 | 0.086 | -0.196 | 0.687 | 0.091 |
| 0.95 | HAP-BLUP-LD_0.15 | 0.310 | 0.087 | -0.191 | 0.687 | 0.091 |
| 0.95 | HAP-BLUP-LD_0.35 | 0.310 | 0.086 | -0.188 | 0.687 | 0.091 |
| 0.95 | HAP-BLUP-LD_0.50 | 0.308 | 0.086 | -0.199 | 0.687 | 0.091 |
| 0.95 | HAP-BLUP-LD_0.65 | 0.309 | 0.086 | -0.197 | 0.687 | 0.091 |
| 0.95 | HAP-BLUP-LD_0.80 | 0.308 | 0.086 | -0.198 | 0.687 | 0.091 |
| 0.50 | H-BLUP | 0.305 | 0.078 | -0.096 | 0.664 | 0.101 |
| 0.50 | HAP-BLUP-LD_0.15 | 0.307 | 0.079 | -0.090 | 0.665 | 0.101 |
| 0.50 | HAP-BLUP-LD_0.35 | 0.306 | 0.078 | -0.089 | 0.664 | 0.102 |
| 0.50 | HAP-BLUP-LD_0.50 | 0.305 | 0.078 | -0.096 | 0.664 | 0.103 |
| 0.50 | HAP-BLUP-LD_0.65 | 0.305 | 0.078 | -0.095 | 0.664 | 0.104 |
| 0.50 | HAP-BLUP-LD_0.80 | 0.305 | 0.078 | -0.096 | 0.664 | 0.105 |

^1^ BLUP = Best Linear Unbiased Prediction; SNP = Single Nucleotide Polymorphisms; A-BLUP = pedigree-based BLUP; H-BLUP = SNP-based BLUP; HAP-BLUP-LD_0.15, HAP-BLUP-LD_0.35, HAP-BLUP-LD_0.50, HAP-BLUP-LD_0.65, HAP-BLUP-LD_0.80 = haplotype-based BLUP using non-clustered SNPs and pseudo-SNPs from haploblocks with linkage disequilibrium thresholds of 0.15, 0.35, 0.50, 0.65, and 0.80, respectively. ^2^ Mean theoretical accuracy. ^3^ Standard deviation for the mean theoretical accuracy.

**Supplementary File 2 Table 6.** Results of genetic and single-step genomic predictions for number of lambs born in Rambouillet sheep using alpha equal to 0.95 or 0.50 to create the genomic relationship matrix.

| **Alpha** | **Method^1^** | **Accuracy** | **Bias** | **Dispersion** | **TA_mean^2^** | **TA_sd^3^** |
| --- | --- | --- | --- | --- | --- | --- |
| none | A-BLUP | 0.148 | 0.001 | -0.397 | 0.562 | 0.076 |
| 0.95 | H-BLUP | 0.202 | 0.005 | -0.357 | 0.598 | 0.058 |
| 0.95 | HAP-BLUP-LD_0.15 | 0.202 | 0.005 | -0.346 | 0.598 | 0.059 |
| 0.95 | HAP-BLUP-LD_0.35 | 0.202 | 0.005 | -0.357 | 0.598 | 0.059 |
| 0.95 | HAP-BLUP-LD_0.50 | 0.203 | 0.005 | -0.354 | 0.598 | 0.059 |
| 0.95 | HAP-BLUP-LD_0.65 | 0.202 | 0.005 | -0.358 | 0.598 | 0.059 |
| 0.95 | HAP-BLUP-LD_0.80 | 0.202 | 0.005 | -0.360 | 0.598 | 0.059 |
| 0.50 | H-BLUP | 0.166 | 0.004 | -0.393 | 0.571 | 0.064 |
| 0.50 | HAP-BLUP-LD_0.15 | 0.166 | 0.004 | -0.385 | 0.572 | 0.064 |
| 0.50 | HAP-BLUP-LD_0.35 | 0.166 | 0.004 | -0.391 | 0.572 | 0.064 |
| 0.50 | HAP-BLUP-LD_0.50 | 0.167 | 0.004 | -0.391 | 0.571 | 0.064 |
| 0.50 | HAP-BLUP-LD_0.65 | 0.166 | 0.004 | -0.394 | 0.571 | 0.064 |
| 0.50 | HAP-BLUP-LD_0.80 | 0.166 | 0.004 | -0.395 | 0.571 | 0.064 |

^1^ BLUP = Best Linear Unbiased Prediction; SNP = Single Nucleotide Polymorphisms; A-BLUP = pedigree-based BLUP; H-BLUP = SNP-based BLUP; HAP-BLUP-LD_0.15, HAP-BLUP-LD_0.35, HAP-BLUP-LD_0.50, HAP-BLUP-LD_0.65, HAP-BLUP-LD_0.80 = haplotype-based BLUP using non-clustered SNPs and pseudo-SNPs from haploblocks with linkage disequilibrium thresholds of 0.15, 0.35, 0.50, 0.65, and 0.80, respectively. ^2^ Mean theoretical accuracy. ^3^ Standard deviation for the mean theoretical accuracy.
