# Supplementary material for: SNP‐ and haplotype‐based single‐step genomic predictions for body weight, wool, and reproductive traits in North American Rambouillet sheep
Source: J Anim Breed Genet. 2022 Nov 21;140(2):216–34. doi: 10.1111/jbg.12748 (PMC10099590; doi:10.1111/jbg.12748)
Supplement: Supplementary file 3 — Appendix S3. [file JBG-140-216-s003.docx]

**SUPPLEMENTARY FILE 3**


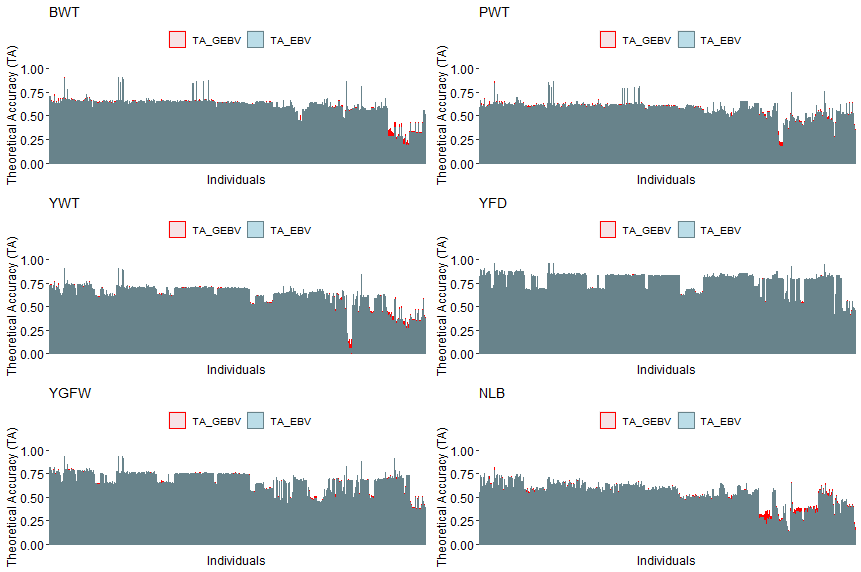


**Supplementary File 3 Figure 1.** Theoretical accuracies for the genomic estimated breeding values fitting single nucleotide polymorphisms (SNP) (TA_GEBV) and estimated breeding values (TA_EBV) for close relatives (progeny or parents) of the genotyped individuals for birth weight (BWT), post-weaning weight (PWT), yearling weight (YWT), yearling fiber diameter (YFD), yearling greasy fleece weight (YGFW), and number of lambs born (NLB). The TA_GEBV and TA_EBV were obtained fitting SNP in the single-step GBLUP (H-BLUP) with alpha equal to 0.95 and pedigree-based BLUP (A-BLUP), respectively.


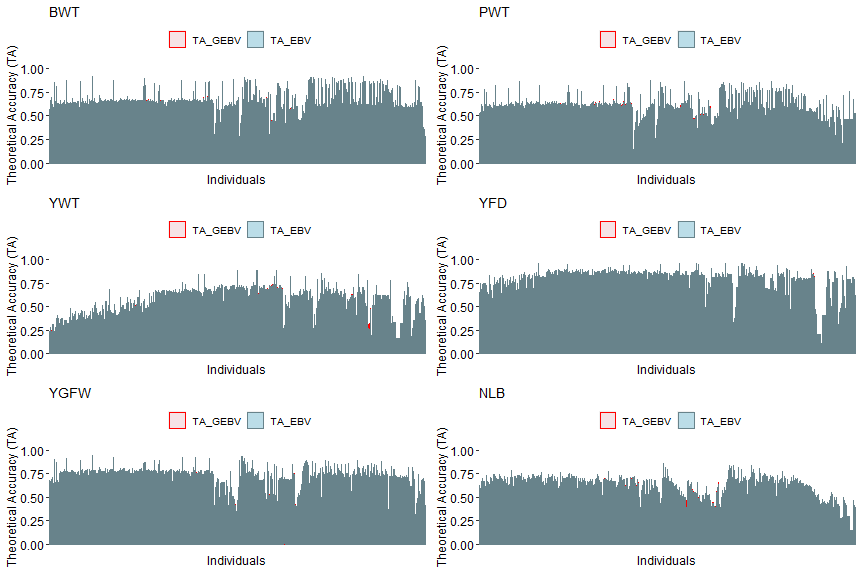


**Supplementary File 3 Figure 2.** Theoretical accuracies for the genomic estimated breeding values fitting single nucleotide polymorphisms (SNP) (TA_GEBV) and estimated breeding values (TA_EBV) for unrelated of the genotyped individuals for birth weight (BWT), post-weaning weight (PWT), yearling weight (YWT), yearling fiber diameter (YFD), yearling greasy fleece weight (YGFW), and number of lambs born (NLB). The TA_GEBV and TA_EBV were obtained fitting SNP in the single-step GBLUP (H-BLUP) with alpha equal to 0.95 and pedigree-based BLUP (A-BLUP), respectively.
