# Supplementary material for: SNP‐ and haplotype‐based single‐step genomic predictions for body weight, wool, and reproductive traits in North American Rambouillet sheep
Source: J Anim Breed Genet. 2022 Nov 21;140(2):216–34. doi: 10.1111/jbg.12748 (PMC10099590; doi:10.1111/jbg.12748)
Supplement: Supplementary file 4 — Appendix S4. [file JBG-140-216-s002.docx]

**SUPPLEMENTARY FILE 4**

**Supplementary File 4 Table 1.** Results of genetic and single-step genomic predictions of birth weight in Rambouillet sheep when considering alpha equal to 0.95 or 0.50 to create the genomic relationship matrix based on the imputed 50K SNP panel.

| **Alpha** | **Method^1^** | **Accuracy** | **Bias** | **Dispersion** | **TA_mean^2^** |
| --- | --- | --- | --- | --- | --- |
| 0.95 | H-BLUP | 0.187 | 0.014 | -0.465 | 0.593(0.052) |
| 0.95 | HAP-BLUP-LD_0.15 | 0.184 | 0.014 | -0.486 | 0.593(0.052) |
| 0.95 | HAP-BLUP-LD_0.35 | 0.183 | 0.015 | -0.497 | 0.593(0.052) |
| 0.95 | HAP-BLUP-LD_0.50 | 0.183 | 0.014 | -0.492 | 0.593(0.052) |
| 0.95 | HAP-BLUP-LD_0.65 | 0.188 | 0.013 | -0.454 | 0.593(0.052) |
| 0.95 | HAP-BLUP-LD_0.80 | 0.187 | 0.014 | -0.463 | 0.593(0.052) |
| 0.50 | H-BLUP | 0.185 | 0.005 | -0.366 | 0.564(0.055) |
| 0.50 | HAP-BLUP-LD_0.15 | 0.184 | 0.005 | -0.383 | 0.564(0.055) |
| 0.50 | HAP-BLUP-LD_0.35 | 0.183 | 0.006 | -0.391 | 0.564(0.055) |
| 0.50 | HAP-BLUP-LD_0.50 | 0.183 | 0.005 | -0.386 | 0.564(0.055) |
| 0.50 | HAP-BLUP-LD_0.65 | 0.186 | 0.005 | -0.358 | 0.564(0.055) |
| 0.50 | HAP-BLUP-LD_0.80 | 0.185 | 0.005 | -0.366 | 0.564(0.055) |

^1^ BLUP = Best Linear Unbiased Prediction, SNP = Single Nucleotide Polymorphisms. A-BLUP = pedigree-based BLUP; H-BLUP = SNP-based BLUP; HAP-BLUP-LD_0.15, HAP-BLUP-LD_0.35, HAP-BLUP-LD_0.50, HAP-BLUP-LD_0.65, HAP-BLUP-LD_0.80 = haplotype-based BLUP using non-clustered SNP and pseudo-SNP from haploblocks with linkage disequilibrium thresholds of 0.15, 0.35, 0.50, 0.65, and 0.80, respectively. ^2^ Mean (standard deviation) theoretical accuracy.

**Supplementary File 4 Table 2.** Results of genetic and single-step genomic predictions of post weaning weight in Rambouillet sheep when considering alpha equal to 0.95 or 0.50 to create the genomic relationship matrix based on the imputed 50K SNP panel.

| **Alpha** | **Method^1^** | **Accuracy** | **Bias** | **Dispersion** | **TA_mean^2^** |
| --- | --- | --- | --- | --- | --- |
| 0.95 | H-BLUP | 0.209 | -0.101 | -0.286 | 0.550(0.056) |
| 0.95 | HAP-BLUP-LD_0.15 | 0.210 | -0.102 | -0.281 | 0.550(0.056) |
| 0.95 | HAP-BLUP-LD_0.35 | 0.210 | -0.101 | -0.281 | 0.550(0.056) |
| 0.95 | HAP-BLUP-LD_0.50 | 0.211 | -0.101 | -0.274 | 0.549(0.056) |
| 0.95 | HAP-BLUP-LD_0.65 | 0.209 | -0.100 | -0.284 | 0.549(0.056) |
| 0.95 | HAP-BLUP-LD_0.80 | 0.209 | -0.100 | -0.284 | 0.550(0.056) |
| 0.50 | H-BLUP | 0.167 | -0.056 | -0.453 | 0.517(0.059) |
| 0.50 | HAP-BLUP-LD_0.15 | 0.168 | -0.056 | -0.448 | 0.517(0.059) |
| 0.50 | HAP-BLUP-LD_0.35 | 0.168 | -0.056 | -0.451 | 0.517(0.059) |
| 0.50 | HAP-BLUP-LD_0.50 | 0.168 | -0.056 | -0.447 | 0.517(0.059) |
| 0.50 | HAP-BLUP-LD_0.65 | 0.167 | -0.055 | -0.453 | 0.517(0.059) |
| 0.50 | HAP-BLUP-LD_0.80 | 0.167 | -0.055 | -0.452 | 0.517(0.059) |

^1^ BLUP = Best Linear Unbiased Prediction; SNP = Single Nucleotide Polymorphisms; A-BLUP = pedigree-based BLUP; H-BLUP = SNP-based BLUP; HAP-BLUP-LD_0.15, HAP-BLUP-LD_0.35, HAP-BLUP-LD_0.50, HAP-BLUP-LD_0.65, HAP-BLUP-LD_0.80 = haplotype-based BLUP using non-clustered SNPs and pseudo-SNPs from haploblocks with linkage disequilibrium thresholds of 0.15, 0.35, 0.50, 0.65, and 0.80, respectively. ^2^ Mean (standard deviation) theoretical accuracy.

**Supplementary File 4 Table 3.** Results of genetic and single-step genomic predictions of yearling weight in Rambouillet sheep when considering alpha equal to 0.95 or 0.50 to create the genomic relationship matrix based on the imputed 50K SNP panel.

| **Alpha** | **Method^1^** | **Accuracy** | **Bias** | **Dispersion** | **TA_mean^2^** |
| --- | --- | --- | --- | --- | --- |
| 0.95 | H-BLUP | 0.299 | -0.081 | 0.957 | 0.652(0.038) |
| 0.95 | HAP-BLUP-LD_0.15 | 0.302 | -0.086 | 0.953 | 0.652(0.038) |
| 0.95 | HAP-BLUP-LD_0.35 | 0.301 | -0.081 | 0.949 | 0.651(0.038) |
| 0.95 | HAP-BLUP-LD_0.50 | 0.298 | -0.084 | 0.954 | 0.651(0.038) |
| 0.95 | HAP-BLUP-LD_0.65 | 0.298 | -0.082 | 0.951 | 0.652(0.038) |
| 0.95 | HAP-BLUP-LD_0.80 | 0.298 | -0.083 | 0.955 | 0.652(0.038) |
| 0.50 | H-BLUP | 0.237 | -0.133 | 0.896 | 0.632(0.040) |
| 0.50 | HAP-BLUP-LD_0.15 | 0.239 | -0.135 | 0.895 | 0.632(0.041) |
| 0.50 | HAP-BLUP-LD_0.35 | 0.238 | -0.134 | 0.891 | 0.631(0.041) |
| 0.50 | HAP-BLUP-LD_0.50 | 0.236 | -0.135 | 0.894 | 0.631(0.040) |
| 0.50 | HAP-BLUP-LD_0.65 | 0.237 | -0.134 | 0.891 | 0.632(0.040) |
| 0.50 | HAP-BLUP-LD_0.80 | 0.237 | -0.134 | 0.894 | 0.632(0.040) |

^1^ BLUP = Best Linear Unbiased Prediction; SNP = Single Nucleotide Polymorphisms; A-BLUP = pedigree-based BLUP; H-BLUP = SNP-based BLUP; HAP-BLUP-LD_0.15, HAP-BLUP-LD_0.35, HAP-BLUP-LD_0.50, HAP-BLUP-LD_0.65, HAP-BLUP-LD_0.80 = haplotype-based BLUP using non-clustered SNPs and pseudo-SNPs from haploblocks with linkage disequilibrium thresholds of 0.15, 0.35, 0.50, 0.65, and 0.80, respectively. ^2^ Mean (standard deviation) theoretical accuracy.

**Supplementary File 4 Table 4.** Results of genetic and single-step genomic predictions of yearling fiber diameter in Rambouillet sheep when considering alpha equal to 0.95 or 0.50 to create the genomic relationship matrix based on the imputed 50K SNP panel.

| **Alpha** | **Method^1^** | **Accuracy** | **Bias** | **Dispersion** | **TA_mean^2^** |
| --- | --- | --- | --- | --- | --- |
| 0.95 | H-BLUP | 0.314 | 0.022 | 0.805 | 0.797(0.087) |
| 0.95 | HAP-BLUP-LD_0.15 | 0.314 | 0.025 | 0.795 | 0.796(0.087) |
| 0.95 | HAP-BLUP-LD_0.35 | 0.314 | 0.024 | 0.801 | 0.796(0.087) |
| 0.95 | HAP-BLUP-LD_0.50 | 0.314 | 0.023 | 0.802 | 0.796(0.087) |
| 0.95 | HAP-BLUP-LD_0.65 | 0.316 | 0.023 | 0.806 | 0.796(0.087) |
| 0.95 | HAP-BLUP-LD_0.80 | 0.316 | 0.022 | 0.809 | 0.797(0.087) |
| 0.50 | H-BLUP | 0.296 | 0.024 | 0.851 | 0.783(0.097) |
| 0.50 | HAP-BLUP-LD_0.15 | 0.295 | 0.025 | 0.839 | 0.782(0.097) |
| 0.50 | HAP-BLUP-LD_0.35 | 0.295 | 0.024 | 0.844 | 0.486(0.155) |
| 0.50 | HAP-BLUP-LD_0.50 | 0.295 | 0.024 | 0.845 | 0.782(0.097) |
| 0.50 | HAP-BLUP-LD_0.65 | 0.297 | 0.024 | 0.848 | 0.782(0.097) |
| 0.50 | HAP-BLUP-LD_0.80 | 0.297 | 0.024 | 0.851 | 0.782(0.097) |

^1^ BLUP = Best Linear Unbiased Prediction; SNP = Single Nucleotide Polymorphisms; A-BLUP = pedigree-based BLUP; H-BLUP = SNP-based BLUP; HAP-BLUP-LD_0.15, HAP-BLUP-LD_0.35, HAP-BLUP-LD_0.50, HAP-BLUP-LD_0.65, HAP-BLUP-LD_0.80 = haplotype-based BLUP using non-clustered SNPs and pseudo-SNPs from haploblocks with linkage disequilibrium thresholds of 0.15, 0.35, 0.50, 0.65, and 0.80, respectively. ^2^ Mean (standard deviation) theoretical accuracy.

**Supplementary File 4 Table 5.** Results of genetic and single-step genomic predictions of yearling greasy fleece weight in Rambouillet sheep when considering alpha equal to 0.95 or 0.50 to create the genomic relationship matrix based on the imputed 50K SNP panel.

| **Alpha** | **Method^1^** | **Accuracy** | **Bias** | **Dispersion** | **TA_mean^2^** |
| --- | --- | --- | --- | --- | --- |
| 0.95 | H-BLUP | 0.422 | 0.079 | 0.28 | 0.696(0.088) |
| 0.95 | HAP-BLUP-LD_0.15 | 0.426 | 0.080 | 0.294 | 0.695(0.088) |
| 0.95 | HAP-BLUP-LD_0.35 | 0.423 | 0.079 | 0.280 | 0.695(0.088) |
| 0.95 | HAP-BLUP-LD_0.50 | 0.422 | 0.08 | 0.279 | 0.695(0.088) |
| 0.95 | HAP-BLUP-LD_0.65 | 0.421 | 0.080 | 0.272 | 0.695(0.088) |
| 0.95 | HAP-BLUP-LD_0.80 | 0.421 | 0.079 | 0.278 | 0.695(0.088) |
| 0.50 | H-BLUP | 0.409 | 0.071 | 0.379 | 0.674(0.098) |
| 0.50 | HAP-BLUP-LD_0.15 | 0.412 | 0.071 | 0.390 | 0.674(0.098) |
| 0.50 | HAP-BLUP-LD_0.35 | 0.410 | 0.071 | 0.382 | 0.674(0.098) |
| 0.50 | HAP-BLUP-LD_0.50 | 0.409 | 0.071 | 0.379 | 0.674(0.098) |
| 0.50 | HAP-BLUP-LD_0.65 | 0.408 | 0.071 | 0.376 | 0.674(0.098) |
| 0.50 | HAP-BLUP-LD_0.80 | 0.408 | 0.071 | 0.379 | 0.674(0.098) |

^1^ BLUP = Best Linear Unbiased Prediction; SNP = Single Nucleotide Polymorphisms; A-BLUP = pedigree-based BLUP; H-BLUP = SNP-based BLUP; HAP-BLUP-LD_0.15, HAP-BLUP-LD_0.35, HAP-BLUP-LD_0.50, HAP-BLUP-LD_0.65, HAP-BLUP-LD_0.80 = haplotype-based BLUP using non-clustered SNPs and pseudo-SNPs from haploblocks with linkage disequilibrium thresholds of 0.15, 0.35, 0.50, 0.65, and 0.80, respectively. ^2^ Mean (standard deviation) theoretical accuracy.

**Supplementary File 4 Table 6.** Results of genetic and single-step genomic predictions of number of lambs born in Rambouillet sheep when considering alpha equal to 0.95 or 0.50 to create the genomic relationship matrix based on the imputed 50K SNP panel.

| **Alpha** | **Method^1^** | **Accuracy** | **Bias** | **Dispersion** | **TA_mean^2^** |
| --- | --- | --- | --- | --- | --- |
| 0.95 | H-BLUP | 0.217 | 0.004 | -0.314 | 0.599(0.058) |
| 0.95 | HAP-BLUP-LD_0.15 | 0.216 | 0.004 | -0.314 | 0.599(0.059) |
| 0.95 | HAP-BLUP-LD_0.35 | 0.216 | 0.004 | -0.319 | 0.598(0.058) |
| 0.95 | HAP-BLUP-LD_0.50 | 0.217 | 0.004 | -0.315 | 0.598(0.058) |
| 0.95 | HAP-BLUP-LD_0.65 | 0.217 | 0.004 | -0.315 | 0.598(0.058) |
| 0.95 | HAP-BLUP-LD_0.80 | 0.217 | 0.004 | -0.313 | 0.598(0.058) |
| 0.50 | H-BLUP | 0.181 | 0.003 | -0.342 | 0.572(0.064) |
| 0.50 | HAP-BLUP-LD_0.15 | 0.180 | 0.003 | -0.342 | 0.572(0.064) |
| 0.50 | HAP-BLUP-LD_0.35 | 0.180 | 0.003 | -0.345 | 0.572(0.064) |
| 0.50 | HAP-BLUP-LD_0.50 | 0.181 | 0.003 | -0.343 | 0.572(0.064) |
| 0.50 | HAP-BLUP-LD_0.65 | 0.18 | 0.003 | -0.344 | 0.572(0.064) |
| 0.50 | HAP-BLUP-LD_0.80 | 0.18 | 0.003 | -0.342 | 0.572(0.064) |

^1^ BLUP = Best Linear Unbiased Prediction; SNP = Single Nucleotide Polymorphisms; A-BLUP = pedigree-based BLUP; H-BLUP = SNP-based BLUP; HAP-BLUP-LD_0.15, HAP-BLUP-LD_0.35, HAP-BLUP-LD_0.50, HAP-BLUP-LD_0.65, HAP-BLUP-LD_0.80 = haplotype-based BLUP using non-clustered SNPs and pseudo-SNPs from haploblocks with linkage disequilibrium thresholds of 0.15, 0.35, 0.50, 0.65, and 0.80, respectively. ^2^ Mean (standard deviation) theoretical accuracy.
